# SPIRE—a software tool for bicontinuous phase recognition: application for plastid cubic membranes

**DOI:** 10.1093/plphys/kiab476

**Published:** 2021-10-18

**Authors:** Tobias M Hain, Michał Bykowski, Matthias Saba, Myfanwy E Evans, Gerd E Schröder-Turk, Łucja Kowalewska

**Affiliations:** 1 Institute of Mathematics, University of Potsdam, Potsdam D-14476, Germany; 2 College of Science, Health, Engineering and Education, Mathematics and Statistics, Murdoch University, Murdoch WA 6150, Australia; 3 Physical Chemistry, Center for Chemistry and Chemical Engineering, Lund University, Lund 22100, Sweden; 4 Department of Plant Anatomy and Cytology, Faculty of Biology, Institute of Experimental Plant Biology and Biotechnology, University of Warsaw, Warsaw, Poland; 5 Adolphe Merkle Institute, University of Fribourg, Fribourg CH-1700, Switzerland; 6 Department of Applied Mathematics, The Australian National University, Research School of Physics, Canberra 2601, Australia

## Abstract

Bicontinuous membranes in cell organelles epitomize nature’s ability to create complex functional nanostructures. Like their synthetic counterparts, these membranes are characterized by continuous membrane sheets draped onto topologically complex saddle-shaped surfaces with a periodic network-like structure. Their structure sizes, (around 50–500 nm), and fluid nature make transmission electron microscopy (TEM) the analysis method of choice to decipher their nanostructural features. Here we present a tool, Surface Projection Image Recognition Environment (SPIRE), to identify bicontinuous structures from TEM sections through interactive identification by comparison to mathematical “nodal surface” models. The prolamellar body (PLB) of plant etioplasts is a bicontinuous membrane structure with a key physiological role in chloroplast biogenesis. However, the determination of its spatial structural features has been held back by the lack of tools enabling the identification and quantitative analysis of symmetric membrane conformations. Using our SPIRE tool, we achieved a robust identification of the bicontinuous diamond surface as the dominant PLB geometry in angiosperm etioplasts in contrast to earlier long-standing assertions in the literature. Our data also provide insights into membrane storage capacities of PLBs with different volume proportions and hint at the limited role of a plastid ribosome localization directly inside the PLB grid for its proper functioning. This represents an important step in understanding their as yet elusive structure–function relationship.

## Teaser

The SPIRE tool facilitates recognition of bicontinuous phases on transmission electron micrographs, which enables in-depth spatial prolamellar body structure analysis from 2D micrographs.

## Introduction

Biological membranes, dynamic yet stable unique assemblies of lipids and proteins, are selective barriers and enzymatically active regions playing a crucial role in orchestrated cells’ functioning. From a structural point of view, they are described mainly as flat sheets or small folded isolated entities called vesicles. Interestingly, in specific cases, almost all types of cellular membranes can form symmetrical, bicontinuous configurations called “cubic membranes” (Almsherqi et al., 2006, 2009). These are characterized by a spatial structure based on a periodic network or labyrinth-like geometry, defined by uninterrupted negatively curved membranes and with high symmetry (often cubic; Luzzati, 1997). These can be modeled by negatively curved surfaces as the spatial model for the bilayer membrane. Note, that herein the phrase “cubic membrane” is used synonymously for any bicontinuous membranes with two membrane-separated aqueous channels and with a cubic or otherwise highly symmetric spatial structure.

Cubic membranes are observed in cells of different organisms, from protozoa to mammals. They can self-organize from almost all types of membranes, including, for example, endoplasmic reticulum, plasma membrane, mitochondria and plastid inner membranes, and inner nuclear membrane (reviewed in Almsherqi et al., 2009). Due to the length scale of such structures with typical periodicities between 50 and 500 nm, our knowledge about cubic membrane arrangements is almost exclusively obtained from electron microscopy data. Note that this is in contrast to bicontinuous soft matter phases, with much smaller periodicity and homogeneous nature of samples, where X-ray and neutron scattering have been traditionally used to identify structures.

Although the highly regular nature of the membrane arrangements has been noted by many authors, structure identification remains difficult, due to a lack of widely available image processing tools for this purpose. As a result, many of these structures have been inaccurately identified as, for example, tubular inclusions, undulating membranes, or cisternal systems (for the review of this issue, see Almsherqi et al., 2006, 2009; Cui et al., 2020), instead of associating them correctly with cubic phases. The deep understanding of the factors governing the formation of such complex amphiphilic arrangements is still not established, partially due to the scattered nature and incorrect annotations of reported data. On the other hand, considerable advancement in recognition of intrinsic and extrinsic factors playing a role in elementary membrane bending and curvature sensing has been made, forming a solid foundation for further studies in understanding how the complex architecture of cubic membranes controls cellular traffic (Kozlov et al., 2014; Jarsch et al., 2016; Lou et al., 2018; Simunovic et al., 2019; Callens et al., 2020).

Recently, there is a growing interest in the possibility of obtaining nature-inspired and, therefore, stable, large length-scaled cubic systems (>50 nm) to develop concepts to tackle different multidisciplinary issues and healthcare problems (reviewed in Mezzenga et al., 2019). The potential application of inducible cubic membranes in plant synthetic biology has also been raised (Sandor et al., 2021). However, such broad interdisciplinary interest in bicontinuous systems has not yet been addressed by recent fundamental research on naturally occurring cubic membranes. Key factors constraining advances in this field are the recognition and spatial analysis of observed membrane arrangements. Such data are crucial to establishing a model system for further biochemical studies and, finally, to discover the shape-dependent role of cubic membranes. This generates a great demand for methods to recognize and measure 3D properties of periodic assemblies.

In terms of topology and geometry, cubic membrane structures can be described using triply periodic minimal surfaces (TPMSs). Minimal surfaces are surfaces that locally minimize their surface area based on some global constraint (e.g. a surface between a given boundary). As a consequence of this minimization, they have a mean curvature of zero at all points on the surface. TPMSs are minimal surfaces with a crystalline, symmetric structure where they repeat in three independent translation directions in space. A wide array of TPMSs has been described mathematically with different crystallographic symmetries. However, three particular TPMSs with cubic symmetry are most commonly observed in biological cubic membranes. Primitive and diamond types were characterized by Schwarz in 1865 and the so-called gyroid recognized by Schoen (1970) almost a hundred years later (1970) (reviewed in Hyde et al., 1996). TPMSs divide inner space into two separated, intertwining yet open channels. In terms of cubic membranes, the presence of such isolated regions of given sizes might have tremendous consequences in constraining molecular motion.

**Figure 1:**
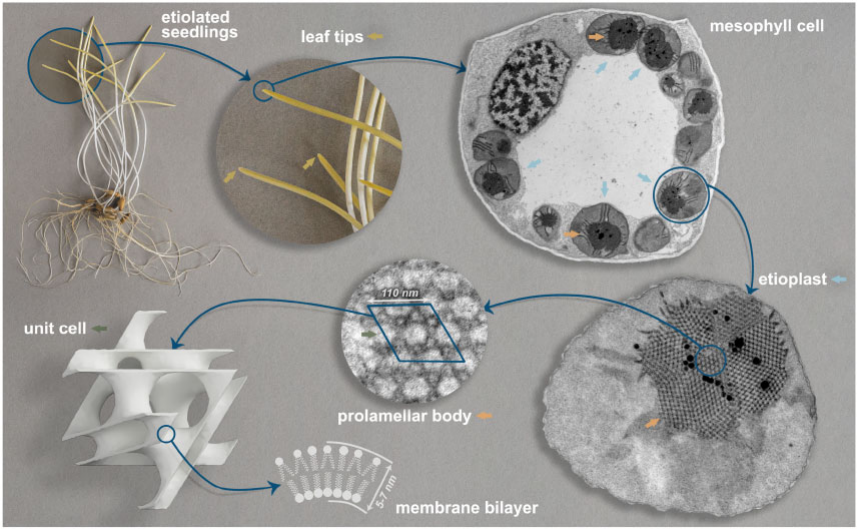
Cellular localization of cubic membrane assembly—PLB in etiolated seedlings of oat. Cubic structure of PLBs located in etiolated seedlings of angiosperms—here exemplified by growing for 7 d in complete darkness. PLBs develop in etioplasts present in developing leaves’ mesophyll cells (yellow parts of the seedling visible above white part of the shoot). The cubic arrangement of the PLB is characterized by a single membrane separating two water channels of different volumes (referred to as being imbalanced, with volume proportions different from 50%) and a relatively small length scale compared to other naturally occurring cubic membranes. Note that recent results indicated that PLB membranes are densely decorated with light-dependent protochlorophyllide oxidoreductase protein, whose role in membrane tubulation has been proven in *in vitro* studies (Floris and Kuhlbrandt, 2021; Nguyen et al., 2021). Apart from marked elements, the presented photographs, and electron micrographs are not shown to scale; mesophyll cell and etioplast are free-form selected manually from TEM images of etiolated oat leaves.

One of the examples of extensively studied cubic membrane assemblies is a prolamellar body (PLB) of plant etioplasts, see Figure  1. The PLB is a direct precursor of the chloroplast thylakoid network, and their lipid–pigment–protein composition shares some similarities (for the review of PLB composition, see Adam et al., 2011; Pribil et al., 2014; Kowalewska et al., 2019). The PLB is considered as a lipid reservoir during tubular–lamellar transition increasing the efficiency of grana formation (Armarego-Marriott et al., 2019; Pipitone et al., 2021). The number of PLB building blocks plays a crucial role in maintaining its structure, including the protochlorophyllide:light-dependent protochlorophyllide oxidoreductase:NADPH complex as well as particular galactolipids and carotenoids (Sperling et al., 1998; Fujii et al., 2019; Bykowski et al., 2020; Cazzonelli et al., 2020; Floris and Kuhlbrandt, 2021; Nguyen et al., 2021). Although the role of light-dependent protochlorophyllide oxidoreductase in membrane tubulation was proven recently using electron cryo-tomography techniques (Floris and Kuhlbrandt, 2021; Nguyen et al., 2021), factors governing the transition of tubular arrangements into cubic configuration remain elusive (Wietrzynski and Engel, 2021). PLBs are rare examples of cubic membranes which can be modeled using imbalanced TPMS, that is, the two channels separated by the membrane are geometrically different such that one has a smaller volume. In relation to the PLB, this means the volumes of the two aqueous channels differ substantially—beyond natural fluctuations—from each other. The smaller channel is a direct precursor of thylakoid lumen of chloroplasts (Kowalewska et al., 2016). In early studies, PLB structures were most frequently referred to as zinc sulfide crystal forms of wurtzite (lonsdaleite) and zincblende type, both based on tetrahedral units forming complex hexagonal networks and as such with hexagonal instead of cubic symmetry. The primitive, face-centered diamond and double diamond cubic membrane types were also proposed (Menke, 1963; Ikeda, 1968; Gunning and Steer, 1975; Landh, 1996). These variable structural annotations were made based on the analyses of randomly cut PLB sections visible in 2D transmission electron microscopy (TEM) micrographs via their comparison with 3D models (physical or rendered) of the mentioned structures. However, even comparing many 2D projections of a 3D structure (TEM specimen) at different viewing angles with an actual 3D model is not directly verifiable and probably led to such inconsistency in the identification of the most abundant spatial PLB configuration.

There are two main methods for analyzing the 3D structure of PLB and other cubic membrane arrangements. Both methods are based on the assumption that cubic membrane structures correspond directly to mathematically well-defined TPMSs. The first approach consists of the visualization of cubic membranes using electron tomography, further segmentation, modeling the periodic arrangement, and finally, its direct comparison to the rendered 3D model of different bicontinuous structures with variable surface parameters and length scales (Chong and Deng, 2012; Demurtas et al., 2015; Kowalewska et al., 2016). Such a method is time, money, and computational power-consuming due to the operation on the 3D objects. It is also limited to the cubic membrane structures of particular length scales. Moreover, it should be stressed that manual segmentation of cubic membranes is complicated, and automated methods, while sufficient to estimate general structural parameters (e.g. channel volume or surface area), fail in terms of precise shape visualization.

Alternatively, the second method is performed using 2D TEM images of cubic membranes and their direct comparison with a simulation of a 2D TPMS projection of given parameters. The idea of the “template matching” method for cubic structure recognition was initially introduced by Mark Mieczkowski and Yuru Deng (Deng and Mieczkowski, 1998; Deng et al., 1999). It was successfully implemented to recognize the surface type of several membrane arrangements, for example, in mitochondria of starved *Chaos carolinensis* (Deng et al., 1999) or chloroplasts of green alga *Zygnema* sp. in the log phase of growth (Zhan et al., 2017) using the developed software called cubic membrane simulation program (QMSP). The QMSP tool enabled the generation of a library of projection images for structures of primitive, double diamond, and gyroid surfaces; the user could manipulate the direction of the projection, number of visualized unit cells (UCs), and thickness of the projected TPMS region. The tool is not publicly available and has limited functionalities in projection scaling, surface types, channel balance, and measurement properties.

Inspired by Deng and Mieczkowski (1998), we introduce Surface Projection Image Recognition Environment (SPIRE), an open-source tool to simulate TEM images of TPMSs. It addresses the aforementioned issues, vastly extends and improves the structure identification process by focusing on interactive matching, and lays the foundation for an automated identification process.

The SPIRE tool focuses on the interactive matching of TEM images, with access to a large range of parameters and metrics of the structure. It is broadly applicable to reliably recognize and analyze structural, spatial properties of bicontinuous arrangements visualized in electron microscopy. A widespread representation of cubic membranes in living organisms highlights the importance of our tool for a large community of biologists. The intuitive SPIRE graphical user interface (GUI) can be used by a broad group of scientists, including those with no explicit knowledge of the geometrical description of cubic structures.

Although we developed SPIRE to investigate cellular cubic membranes visualized in TEM images, it is also a suitable tool for analyzing microscopy images of any highly symmetric arrangement such as cuboids or polymer assemblies. In the latter, we see similar geometries to those found in the biological cubic membranes (Bates, 2005; Kirkensgaard et al., 2011; Han et al., 2020) or cubic rod packings (O’Keeffe et al., 2001). The latter is used to model the keratin microstructure in skin cells, a geometry that is closely related to—and likely coexistent with—a gyroid surface (Norlen and Al-Amoudi, 2004; Evans and Hyde, 2011; Evans and Roth, 2014). Synthetic cubic structures are mainly analyzed using scattering methods, but in nonuniform samples, additional microscopy analyses are required.

This article first describes the main concepts and algorithms of the software, followed by a detailed walkthrough of a structure identification process using the PLB arrangements in etiolated seedlings as an example.

## Results

### Numerical procedures

**Figure 2:**
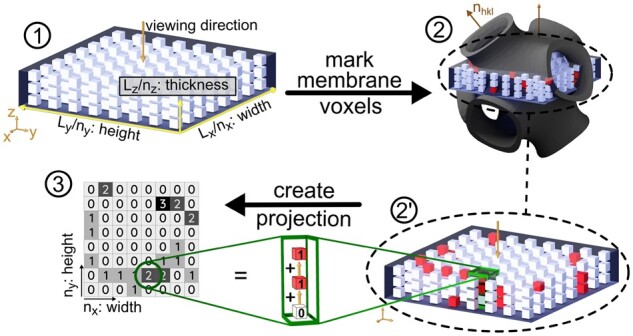
The basic steps in the process of computing a planar projection (1) Schematic representation of the rectangular simulation box (called slice) with the dimensions ($L_{x},\;L_{y},\;L_{z}$), filled with a grid of $n_{x}\cdot n_{y}\cdot n_{z}$ voxels of size $\left(L_{x}/n_{x},\;L_{y}/n_{y},\;L_{z}/n_{z}\right)$. The viewing direction is perpendicular to the $L_{x}L_{y}$ plane. Note that the space between voxels is just for visualization and is not existent in the simulation. (2) All voxels are evaluated using the mathematical model of the surface structure: the slice is superimposed in the oriented membrane structure; a voxel is marked if located within a membrane (2′) The projection is computed by adding the values of voxels with identical x and y coordinates, that is, all voxels congruent in viewing direction. Each marked voxel is valued as “1,” whereas unmarked voxels do not contribute (3). The simulated planar projection, a pixel image where each pixel brightness holds the number of marked, thus membrane, voxels. Its resolution is determined by the number of voxels $\left(n_{x},\;n_{y}\right)$ in the initial slice.

Figure  2 illustrates the major steps in the simulation process used in SPIRE: the simulation of TEM images of ultrathin sections of biological samples, which are essentially planar projections, thus 2D images, of the 3D structures inside the sample. The TEM image of a 3D structure encodes the amount of beam attenuating material along its path that generates the microscope image: whereas dark regions in the image represent areas of a large amount of material along the beam path, brighter regions present less material.

To simulate those projections, a model of the sample is created by using one or multiple membranes, modeled as minimal or negatively curved surfaces. A discrete grid of points, where each point is either marked as “attenuating,” or not marked, that is “translucent,” is then used to compute the planar projection. In this section, details on the underlying processes and methods are presented.

### Mathematical modeling of bicontinous membranes

The so-called nodal representation is used to describe membrane geometries. In this model, the true TPMSs are approximated by implicit functions $f\left(x,y,z\right):E^{3}\to R$, the so-called nodal functions (von Schnering and Nesper, 1991; Klinowski et al., 1996). Surfaces are then defined using so-called level set parametrization: the surface is the set of all points where $f\left(x,y,z\right)=c$, with c being an arbitrary constant. Different values of c yield different surfaces.

SPIRE contains nodal representations for several surfaces as well as rod packings. Three surfaces with cubic symmetries are included: the gyroid, diamond, and primitive surface. The nodal representations of the latter are taken from von Schnering and Nesper (1991). We computed the nodal representation of the lonsdaleite (“hexagonal diamond”) surface from its corresponding spacegroup (SG194) and its structure factor (similar to Wohlgemuth et al., 2001, and references therein). The leading term only representation reads

$$f\left(x,y,z\right)=-\cos\left(2Z\right)+\cos\left(X\right)+\cos\left(Y\right)+\cos\left(X-Y\right)+\sin\left(Z\right)\left[-\sin\left(X\right)+\sin\left(Y\right)+\sin\left(X-Y\right)\right]$$

with

$$\left(X,Y,Z\right)=2\pi A^{-1}.{\left(x,y,z\right)}^{⊺}$$

and **A** being the matrix comprising the three canonical lattice vectors of a structure with hexagonal symmetry: $a_{1}={\left(1,0,0\right)}^{⊺}$, $a_{2}={\left(-\frac{1}{2},\frac{\sqrt{3}}{2},0\right)}^{⊺}$, $a_{3}={\left(0,0,\sqrt{\frac{8}{3}}\right)}^{⊺}$ and the dot denoting the matrix product (see section “Crystallographic nature of highly symmetricmembranes” for more details). Note, that due to their repeating nature, all representations of triply periodic surfaces can be expressed solely using periodic trigonometric functions. Although the presented formula yields a surface with a topology and geometry equivalent to the lonsdaleite structure, it is not a minimal surface. For the implementation in SPIRE, we therefore used a numerically optimized version. A triangulated, minimal lonsdaleite surface was created using an input file by Ken Brakke for the surface evolver (Brakke, 1992). A voxelized version of the surface was created by marking points on a rectangular grid as “1” on one side of the surface and “−1” on the other side. This discrete, 3D test function is approximated by a Fourier series *f(x,y,z)*. The latter has a root everywhere on the surface, thus *f(x,y,z)* is a nodal representation of the surface. The same numerical protocol was used to compute nodal representations of two cubic rod packings, namely the β-Mn and Σ^+^ rod packings. The original input was generated by placing cylinders of a given radius along the invariant axes of the rods, as described in O'Keeffe et al. (2001). Note that for convenience, in this implementation, instead of using the canonical choice of lattice vectors for a hexagonal symmetry, we use orthogonal lattice vectors with a rhomboidal symmetry. Please refer to Supplemental Figure S1 and Supplemental Table S1 in the supplementary material for detailed information.

All TPMSs divide space into two intertwining channels. Here, the nodal representation is chosen such as $f\left(x,y,z\right)=0$ yields the balanced case: the membrane separates two channels of equal volume. Membranes at $f\left(x,y,z\right)=c$ with arbitrary c, separate two channels with unequal volumes. The constant c is thus a measure of the proportion of volumes of the two channels. SPIRE allows to choose the position of the membrane either based on the constant c (“level set”) or the volume proportion of the two channels. Note, however, that only for membranes where $f\left(x,y,z,\right)=0$ the nodal representation does approximate a true minimal surface! Whereas the surface *f(x,y,z)* = 0 will converge toward the true minimal surface by adding more terms to the series expansion, this is not the case for surfaces with *f(x,y,z)* = c where c0. The latter, however, are topologically equivalent (within a symmetric interval of c values) nonminimal, triply periodic surfaces.

Obtained models can only be matched to actual samples within a margin of error due to various reasons such as quality of TEM images, fluctuations in the biological sample, etc. Once matched, however, the model is intrinsically well defined and exact, which means all model parameters are without error margins. Only when using the model to describe the actual sample variability error margins are relevant.

### Discretization of structure models

For the discretization of the structure models, an approach combining level sets of the nodal representations with a distance map is employed as follows. The process begins by computing the value of the nodal representation $f\left(p_{x},p_{y},p_{z}\right)$ for each voxel at position $\left(p_{x},p_{y},p_{z}\right)$ in the simulation box. As shown in Figure  2, a voxel is marked, if c-λ f<, $\;f\left(p_{x},p_{y},p_{z}\right)$<+λ, where λ is a constant with the meaning of the width of the membrane. In a naive implementation, visually speaking a voxel is marked if it is located within the space bounded by the two level-set surfaces given by $f\left(x,y,z\right)=c+\lambda$ and $f\left(x,y,z\right)=c-\lambda$. Note that these two surfaces bounding the membrane are not necessarily parallel and thus would create a membrane with varying width. To resolve this issue, the following procedure is used: the parameter λ is chosen very small, such that ideally the resulting membrane width is only a single voxel. A so-called Euclidean distance map (EDM) (Felzenszwalb and Huttenlocher, 2012) is then computed. This function $\mathrm{EDM}\left(p_{x},p_{y},p_{z}\right)$ assigns each voxel in the slice the value of the distance (in length units) of the current voxel to the closest marked one, thus the closest membrane voxel. All voxels inside one channel are assigned a positive distance, whereas voxels in the other channel have negative distances to the membrane. The membrane with the desired width is then obtained by marking all voxels where the value $\mathrm{EDM}\left(p_{x},p_{y},p_{z}\right)$ is smaller than or equal to half of the membrane width.

Although less common, more complicated geometries such as double bilayer membranes occur in nature (Deng and Mieczkowski, 1998). To model such systems, SPIRE allows for multiple membranes. The positions of additional membranes are given as a distance to the initial, positioned by the level-set membrane, whereas additional parameters control the width of each membrane (see Supplemental Figure S2). Voxels of additional membranes are marked using the EDM computed before: for each membrane i of width $w_{i}$ and distance $d_{i}$ all voxels for which $d_{i}-\left(w_{i}/2\right)\;\mathrm{EDM}\left(p_{x},p_{y},p_{z}\right)\;d_{i}+\left(w_{i}/2\right)$ holds are marked.

Whereas a single bilayer membrane separates space into two channels, each additional membrane will add a further domain (called “channel” in the software; meaning the space between two parallel membranes). Since membranes have a finite width and thus a volume, this software internally considers the latter as channels, too. Channels are labeled with increasing integers starting at “1” with the innermost channel (containing the center of the UC), the innermost membrane then has the channel number “2,” etc. Also see Supplemental Figure S2 for more information.

So far, only systems related to the double “gyroid” (or other “double phases”) where a membrane (modeled as absorbing) separates two aqueous channels (modeled as translucent) were considered. Several examples in nature, such as a gyroid surface in the wing scales of the butterfly (Schroder-Turk et al., 2011) have been reported with a single surface separating two intertwining channels, where one of the two is filled by a solid material. To account for such systems, the software allows to mark all voxels inside a channel, thus models it as opaque.

### Crystallographic nature of highly symmetric membranes

After introducing the mathematical description of the TPMSs and their geometries, this description is now used to create synthetic images corresponding to the membrane structure in voxelized (3D pixel) form.

The simulation process starts with the initialization of the simulation box, a cuboid with dimensions $L_{x}$, $L_{y},$ and $L_{z}$, where each edge is aligned with its corresponding axes of the canonical base, see Figure  2. The simulation box will be called “slice” and represents the region of interest ($L_{x}$ and $L_{y}$) as well as physical thickness ($L_{z}$) of the ultrathin section of tissue captured by the TEM image, and will be filled with a discretized version of the membrane structure. To store the latter, the simulation box is fitted with a regular, rectangular grid of a total of $n_{x}\cdot n_{y}\cdot n_{z}$ sites, see part 1 in Figure  2. On each site, a small cuboid with dimensions (dx, dy, dz), a so-called voxel, is placed, such as there is no overlap or space between two adjacent voxels. Each of these voxels can be “unmarked,” that is, translucent, or “marked,” that is, opaque. In Figure  2, this property is represented by the color “white” and “red” and will be assigned in a later step.

The slice dimension $\left(L_{x},L_{y},L_{z}\right)$ is an important parameter to match the simulated projection to the size of the TEM image. That is, the slice dimensions should be chosen to correspond to the size of the TEM image of the section considered. The number of voxels in the slice can be tuned by providing the number of voxels in $x\;\left(n_{x}\right)$ and $z\;\left(n_{z}\right)$ direction. To obtain voxels with a square footprint $\left(dx\approx dy\right)$ the number of voxels in y direction is then computed to $n_{y}=L_{x}/L_{y}\cdot n_{x}$ (or nearest integer). The voxel dimensions are computed automatically. The number of voxels determines the resolution and hence the quality of the projection, in line with the resolution of the TEM image.

**Figure 3:**
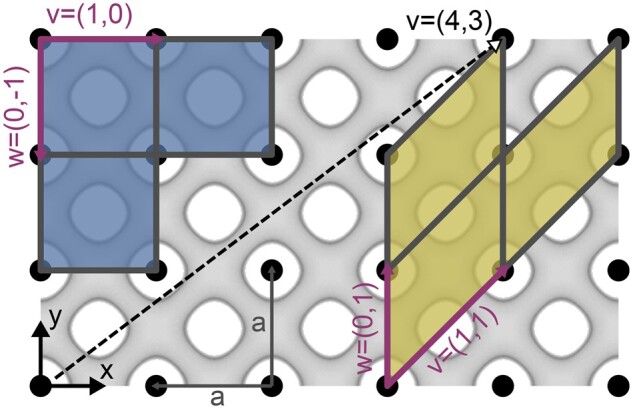
The definition of a UC and its base vectors. An example of a 2D, periodic structure with two choices of lattice vectors and resulting UCs: the fundamental UC (smallest UC with least amount of information to fully replicate the infinite structure while being cubic or rectangular) and an inclination UC (see main text for definition). The black dots are a choice of lattice points and represent geometrically identical, since repeating locations in the structure. The choice of lattice vectors and thus UCs is arbitrary and the size of the UC depends on the choice of lattice vectors: for $v=\left(1,0\right)$ and $w=\left(0,-1\right)$ the UC is square with an edge length of *a* (lattice constant), whereas for $v=\left(1,1\right)$ and $w=\left(0,1\right)$ the UC is a parallelogram with edge lengths $\left|v\right|=\sqrt{2}a$ and $\left|w\right|=a$. The dashed vector v demonstrates that lattice vectors (and thus the UC) can get very long for odd directions.

The software focuses on the modeling of periodic membrane structures. The latter is characterized by the fact that the entire information of a periodic structure is stored in a translational UC and three replication directions, called lattice vectors u, v, and w. We conveniently choose these vectors as the bounding edges of the UC (see Figure  3), although other choices exist and may be more fitting for different purposes. As a consequence, the size and shape of the UC is, apart from the structure itself, solely determined by a choice of the three lattice vectors u, v, and w, that is the replication directions. An infinitely large, continuous structure can be constructed by repeating the UC along the replication directions. Due to this repeating nature, points with identical geometry but different spatial coordinates, here called lattice points, can be identified. The black dots in Figure  3 represent a possible choice of lattice points.

Given a periodic structure, there is no unique choice of a UC: the lattice vectors and thus the UC can be chosen arbitrarily. We introduce a distinct choice of the UC, the herein called fundamental UC. The latter is the cubic (for the surfaces with cubic symmetries, for example, the primitive, diamond, and gyroid surface) or rectangular (for rectangular symmetries, as the Lonsdaleite) UC which among all choices has the smallest volume and content possible (in Figure  3 exactly one lattice point) while still containing all information needed to reproduce the entire structure. In the case of cubic or rectangular TPMSs the lattice vectors u, v, and w, with $\left|u\right|=c$, $\left|v\right|=a$ and $\left|w\right|=b$, of the fundamental UC can be conveniently aligned with the Cartesian coordinate axes x, y, and z. Any UC with a different shape or choice of lattice vectors, especially rotated versions of the fundamental UC, will herein be called inclination UC. The Supplemental Figure S3 lists all fundamental UCs of the structures built into the software.

Whereas in real samples the orientations of the TPMS structures are in most cases random and thus unknown (there are cases, where structures grown onto substrates may exhibit preferred orientations, see Winter et al., 2015; Yoshioka et al., 2014), simulations provide the ability to generate projections with identified orientations. The fixed viewing direction onto the sample is reflected in SPIRE by fixing the viewing direction on to the 3D slice arbitrarily but conveniently to the negative z-direction, that is, perpendicular to the $L_{x}L_{y}$ plane of the slice, as shown in panel 1 in Figure  2.

**Figure 4:**
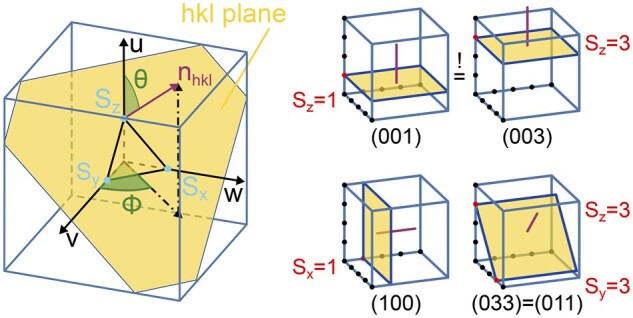
Definition of Miller indices and orientation denotation (left) Formally h,k,and l are defined by $h=p/S_{x}$, $k=p/S_{y}$ and $l=p/S_{z}$ where p is the smallest integer, for which $\left(\mathrm{hkl}\right)$ is a triplet of integers without a common divisor. $S_{x},\;S_{y},\;{\mathrm{and}\;S}_{z}$ are the points, given in terms of the lattice vectors v, w, and u, where the plane, denoted by the Miller indices $\left(\mathrm{hkl}\right)$, intersects the x, y, and z axes (for detailed description of Miller indices principles, see Kittel, 2004). Thus the Miller indices define a plane fixed by three points in space. An index value of 0 means the plane is parallel to the respective axis. For structures with cubic or rectangular fundamental UCs (i.e. all structures implemented in the tool), the normal vector $n_{\mathrm{hkl}}$ on the $\left(\mathrm{hkl}\right)$ plane—and thus a direction—can be written in terms of the Miller indices: $n_{\mathrm{hkl}}={\left(\left(1/a\right)\cdot h,\left(1/b\right)\cdot k,\left(1/c\right)\cdot l\right)}^{⊺}$. The polar angle Φ is the angle between the x-axis and the projection of $n_{\mathrm{hkl}}$ onto the xy-plane, the azimuthal angle Θ is the angle between the z-axis and $n_{\mathrm{hkl}}$. (right). Examples of planes denoted by different values of Miller indices. The red line denotes the normal vector on the plane, whose coordinates, in the case of cubic symmetry, are given by the Miller indices. The black dots denote the coordinate grid spanned by the lattice vectors; each dot represents a symmetrically equivalent position. As a result, although at different positions and with different Miller indices, the two planes in the upper row are equivalent.

The orientation of the membrane structure within the slice is described using the so-called Miller indices $\left(\mathrm{hkl}\right)\;$(Kittel, 2004), a triplet of integer numbers denoting the orientation of a lattice plane and its normal vector $n_{\mathrm{hkl}}$, see Figure  4 for more details. Note that due to the choice of using a rectangular fundamental UC for cubic structures and the hexagonal Lonsdaleite alike, the $\left(\mathrm{hkl}\right)$ values do not correspond to the expected direction in the crystallographic convention and extra care needs to be taken when specifying the inclination. Supplemental Table S1 lists the exact dimensions and choice of lattice vectors of all fundamental UCs in the software. Internally, the orientation of the normal vector given as Miller indices is converted in two angles, the polar angle Φ, and the azimuthal angle Θ, as defined in Figure  4.

In SPIRE, the orientation of the fundamental UC, where v, w, and u align with the coordinate axes, is assigned the “neutral” orientation $\left(001\right)$ with $n_{001}$=z, that is, the orientational normal vector $n_{\mathrm{hkl}}$ aligns with the positive z direction. The two orientation angles then are Φ=0 and Θ=0.

**Figure 5:**
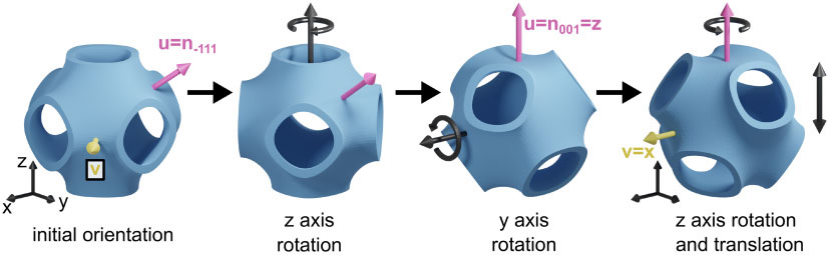
Orienting the membrane structure in the virtual sample. To simulate a projection with the desired viewing angle, the structure needs to be aligned accordingly inside the slice. Starting at the orientation of the fundamental UC, the structure is first rotated around the z-axis by the polar angle Φ, rotating the vector u into the yz-plane, and subsequently around the y-axis by the azimuthal angle Θ, aligning u with the z-axis and thus the viewing direction. The last rotation around the z-axis aligns the in-plane vector v with the x-axis, such that the inclination UC has minimal volume given the normal vector $n_{\mathrm{hkl}}$. In a last step, the structure can be translated along the z-axis to choose the termination.

In the software, a triplet of Miller indices indicates the desired viewing angle on the structure as a vector $n_{\mathrm{hkl}}$. However, since the viewing angle in the tool is fixed to the z axis, the vector $n_{\mathrm{hkl}}$—and with it the structure—needs to be aligned with the z-axis, as described in Figure  5.

After the alignment, the structure can still be rotated around the z axis without changing the orientation determined by $n_{\mathrm{hkl}}$. This remaining rotation is related to the choice of in-plane base vectors v and w. The software first chooses the two lattice vectors v and w in the plane denoted by $\left(\mathrm{hkl}\right)$, and thus perpendicular to $n_{\mathrm{hkl}}$, so that both vectors are as short and as orthogonal to each other as possible (see the blue cell in comparison with the yellow one in Figure  3). Then the surface is rotated around the z-axis until the longer of the two vectors v or w is aligned with the x-axis. Since the lattice in the plane, that is, the pattern of the points in Figure  3, is dependent on the orientation of the plane, the choice of the base vectors differs for each choice of viewing directions $n_{\mathrm{hkl}}$.

Note that for odd combinations of large Miller indices, the UC vectors v, w, and u—and with it, the inclination UC—can get very large (many multiples of the size of the fundamental UC). Figure  3 shows an example vector v, which is long compared to the size of the fundamental UC. This behavior directly translates to three dimensions.

Having the orientation fixed, the last step is to fix the translational degrees of freedom. This can be imagined as the oriented slice being a stencil, cutting a rectangular piece out of the infinite, periodic membrane structure at different locations. Moving a stencil along a periodic structure does not substantially change its content, given the stencil is at least the size of an inclination UC: the slice will in most cases contain one or multiple copies of the UC. As most biological samples will be ultra-thin slices, that is, have a much larger base than thickness, this caveat is met in most cases in the v and w direction of the sample, that is, its width and height, but not the u direction, the viewing direction. Thus, whereas translating the slice through the membrane structure in v and w direction does barely affect the projection, moving it along the u direction will impact the projection substantially, since different parts of the structure are contained in the slice.

SPIRE always centers the slice around the origin, however, allows translation along the normal vector $n_{\mathrm{hkl}}$. In the case of a slice thickness smaller than the size of the inclination UC in the normal direction, this provides the ability to scan through different parts of the UC.

### Synthetic microscopy images as projections of the discretized membrane model

The planar projection of a virtual membrane structure is a pixel image, thus an array of $n_{x}\times n_{y}$ pixels, each with a value indicating the brightness of the pixel. In the biological sample, a dark pixel in a TEM image indicates that much of the brightness of the incident beam has been lost due to a large amount of attenuation with matter, that is, much electron-dense material is located along the path of the beam corresponding to that particular pixel.

This behavior is imitated in SPIRE: each voxel marked as being inside a membrane is assigned a numerical value of “1,” whereas all unmarked pixels, thus representing aqueous phases, are assigned a value of “0.” A value of “1” means that a membrane, therefore attenuating material, is located at that voxel. Adding all voxel values along a given direction yields the total amount of material interaction along that particular path. The values for each pixel at position $\left(i,j\right)$ are computed by choosing a path along the viewing direction, thus normal to the $L_{x}L_{y}$ plane, where the path is located such that it intersects all voxels with identical x and y positions at $p_{x}=i\cdot dx$ and $p_{y}=j\cdot dy$, as shown in Figure  2. As a result, the final resolution of the projection is determined by the resolution of the voxel grid in the simulation box.

To avoid different brightnesses of projections due to varying numbers of voxels, all pixel values in the planar projection images are rescaled to an integer in the range between 0 and 255. This scaling allows for a linear or a logarithmic scale, which depending on the membrane structure may reveal more details in the projection.

### Additional structural properties

On demand, the software can compute the channel volume, the surface area of the membranes, and the percolation threshold of all channels as described below.

#### Channel volume

Each voxel occupies a volume of $V_{v}=dx\cdot dy\cdot dz$, thus the total volume of a channel is just the number of all voxels associated to that channel multiplied with the volume of a single voxel. While there are discretization errors, these are small and decay quickly when voxel sizes are small.

#### Membrane surface area

The surface area of the membranes is computed using a triangulation of the membrane surface. The triangulation is computed by an algorithm called “Advancing front surface reconstruction,” implemented in the computational geometry algorithms library (CGAL) (Da and Cohen-Steiner, 2020; The CGAL Project, 2020), applied to the surface voxels of the membranes. The total surface area of the membrane is the sum of the areas of all triangles in the triangulation. The quality of the approximation of the membrane surface by the triangulation, and thus the accuracy of the surface area, increases with an increasing number of voxels in the simulation box.

#### Percolation threshold

Percolation theory is an area in mathematics, statistical physics, and material science considering basic global connectivity properties of networks and graphs (Stauffer and Aharony, 1992). A network is said to percolate if a path through this network from a defined starting and end point can be found. Removing elements of such a percolating network can cause the connecting path to be cut and thus renders the network nonpercolating.

This software uses percolation analysis to compute the percolation threshold of a channel, that is the maximum diameter of a body (e.g. a sphere or molecule) which can move freely through a channel of the entire structure without getting stuck at narrow passages (Mickel et al., 2008). This measurement is found by increasing the width of the membranes enclosing a channel step by step and checking if the channel is still percolating. The width at which the channel stops to percolate is called the percolation threshold, and denotes the most narrow diameter in the channel. A Hoshen–Kopelmann (Hoshen and Kopelman, 1976) algorithm is used to perform a cluster analysis of all voxels in a channel, that is, all connected nodes of the network are grouped into a single object. The channel is percolating if only a single cluster is found during said analysis, that is each node (and thus voxel) in the channel can be reached from any other node in the same channel. A number of two or more clusters means that the channel has been separated.

## Identification of the structure of prolamellar bodies

### Structure identification process

Here, we present the essential SPIRE features exemplified by matching TEM images of etioplast PLBs with software-generated projections. A video tutorial is provided, proposing an efficient workflow to recognize surface types with proper structural parameters visible on TEM images (http://chloroplast.pl/spire). The appearance of a 3D structure in a 2D projection depends mostly on (1) the scale of a structure regarding its magnification, (2) the thickness of the visualized section, and (3) the orientation of a structure in the TEM sample. We discuss how to use these parameters for proper and efficient matching.

Since the size of the TEM sample is known, the first parameters which can easily be fixed to start the matching are the slice height, width, and thickness. While the height and width only extend or reduce the size of the simulated sample, the thickness does influence the projection, as visualized in Supplemental Figure S4. Two examples of plastid cubic membranes varying in UC size and volume proportion of the two aqueous channels are provided: a gyroid membrane with a large UC and balanced channel volumes present in *Zygnema* sp. chloroplasts (Zhan et al., 2017) and a diamond membrane with a relatively small UC and imbalanced channel volumes found in runner bean (*Phaseolus coccineus*) etioplasts (Kowalewska et al., 2016). Drastic differences in the image characteristics point to the crucial role of the proper identification of scale and channel volume proportion of observed structures. All these properties can be easily calculated directly from the TEM micrographs using standard image analysis tools (e.g. ImageJ).

The next step is to make an initial (educated) guess for the structure type and orientation. A small gallery of all implemented surface types of different UC scales, volume proportions, and orientations are presented in Supplemental Figure S5, which can be used to facilitate this step. Using the bulk creation functions in SPIRE, a user can also create their own, more refined, and suitable galleries to provide better options for an initial guess. In the next step, the user can finely tune all parameters—with direct visual feedback—to further match the simulated projection to the TEM image.

**Figure 6:**
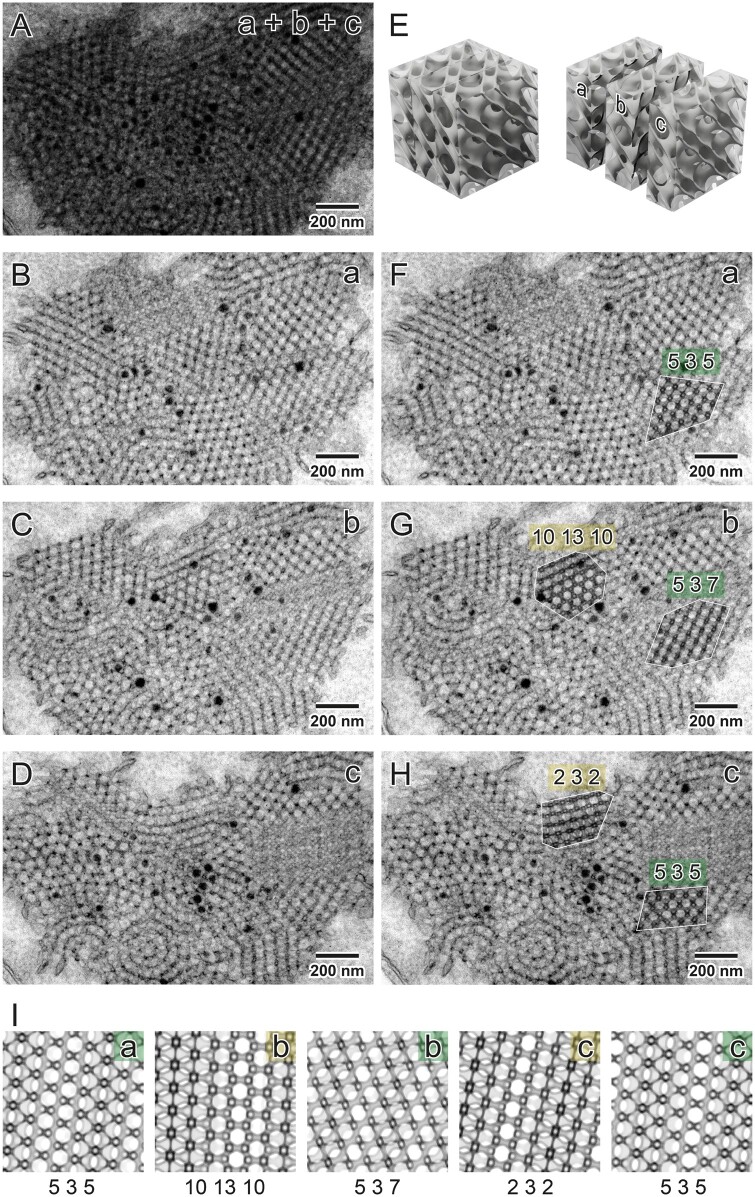
Surface type recognition in particular regions of oat PLB shown in serial TEM sections. Superimposed TEM images (a + b + c) showing PLB projections of a given thickness (70 nm) simulating a thick TEM specimen (210 nm) (A). Serial sectioning of a leaf sample enables visualization of subsequent regions of the PLB cubic structure (B–D); see an exemplary 3D model of the cubic surface presenting the idea of parallel cutting of the specimen block (E). In principle, in selected regions (marked with yellow and green) of subsequent slices (a–c) matched projections should be the same (identical $\left(\mathrm{hkl}\right)$ values) but localized in the different depth of the structure. Such expected depth shift is observed in recognized projections (I) of a diamond surface type; however, matched projections are similar but not always identical in subsequent slices. Such an effect is probably due to the thin TEM sample’s warping during its visualization in the TEM chamber. Accuracy of projection matching is confirmed by superposition of computed projection and TEM image using multiply blend mode (F–H; regions marked with white border); all parameters used to generate projections are listed in Supplemental Table S1.

If the slice thickness is not equal to the size of the inclination UC, that is, it contains a fraction of an inclination UC (the UC in orientation), the projection differs depending on which parts of the inclination UC are contained in the sample. In the software, the UC region is chosen by the slice position parameter. Figure  6 shows three serial sections of the same PLB structure. Selected regions marked with different colors are matched with the following slices, taking into account the slice’s progressing position. The slight inaccuracies in the $\left(\mathrm{hkl}\right)$ values for the same regions in the subsequent slices are probably due to the sample warping during its visualization in TEM; note that neither the UC size nor the channel volume proportion was disturbed.

**Figure 7:**
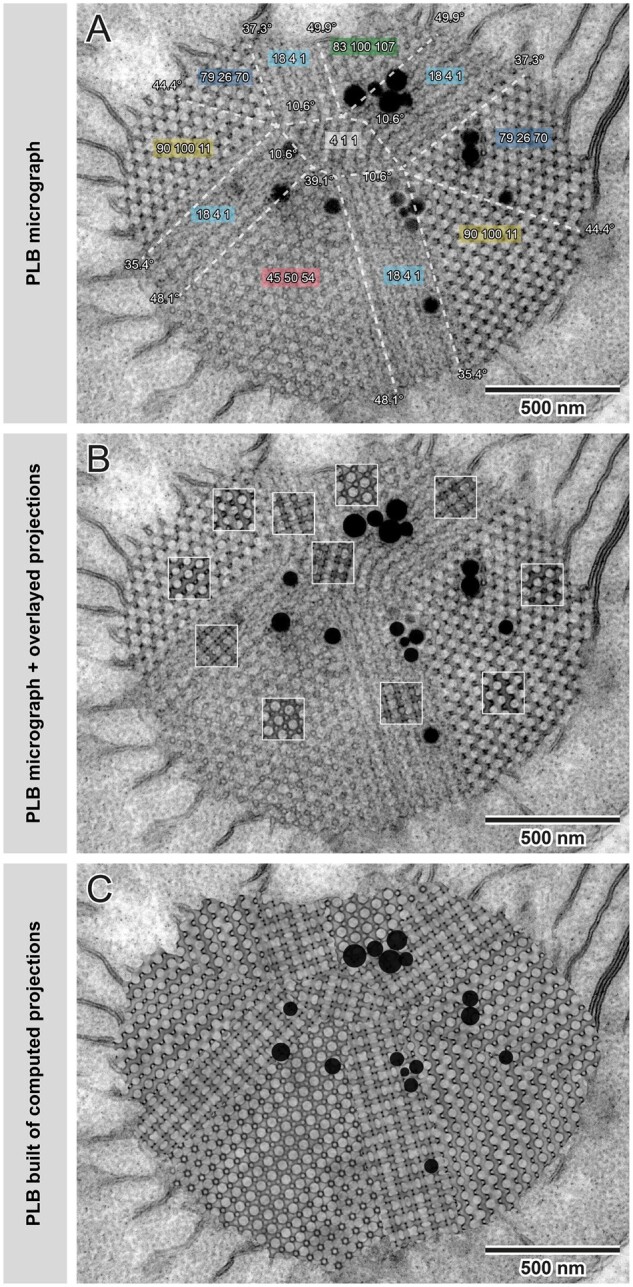
Construction of simulated oat PLB image built of computed projections. In many cases, PLBs and other naturally occurring cubic membranes are composed of several connected regions of bicontinuous surfaces at different orientations, forming “polycrystalline” arrangements (A). For a high confidence identification of the surface type, all different regions should be matched and analyzed. (A; same color indicates identical projections visible in PLB regions connected at different angles). To confirm the consistency of a match, the simulated projections can be superimposed on top of the TEM images (B; regions marked with white squares). To visualize the high accuracy of matches, we constructed an entirely simulated PLB built of particular computed projections (C) connected in the areas marked with white dashed lines visible on (A). Note that random noise was overlaid on computed projections to simulate the typical appearance of the TEM image; black dots added on top of composed projections (C) indicate the position of plastoglobules visible on the TEM micrograph (A, B); all parameters used to generate projections are listed in Supplemental Table S1.

If possible, the identification of the structure should be confirmed by performing the matching procedure on several different regions of the sample with different orientations. These might be taken from different images or from a single image of a polycrystalline sample. PLBs often have a polycrystalline-like structure providing views of several projections of different orientations within one etioplast on a single micrograph; for details see Figure  7.

The user can directly extract different structural features of identified surfaces from the measurement tab of SPIRE. Therefore, it is possible to automatically calculate variable 3D features of a recognized surface based on a 2D TEM image of the structure. Such information is particularly valuable from a biological point of view. Due to the lack of control over the direction of cubic membrane sectioning during sample preparation, recognition of surface type is based on different $\left(\mathrm{hkl}\right)$ projections. Calculations of channel diameters and UC sizes from 2D data are reliable only in cases of specific projections and in terms of primitive surface type also only in the exact depth of the slice. Therefore, the measurement functions of SPIRE, enabling calculations of the 3D features of the recognized structure, bring reliable information about, for example, the membrane area, the volume of the aqueous channels, and the penetrability of the network by molecules of a given sizes; see percolation limit definition above.

### Diamond as the predominant geometry in angiosperm PLBs

**Figure 8:**
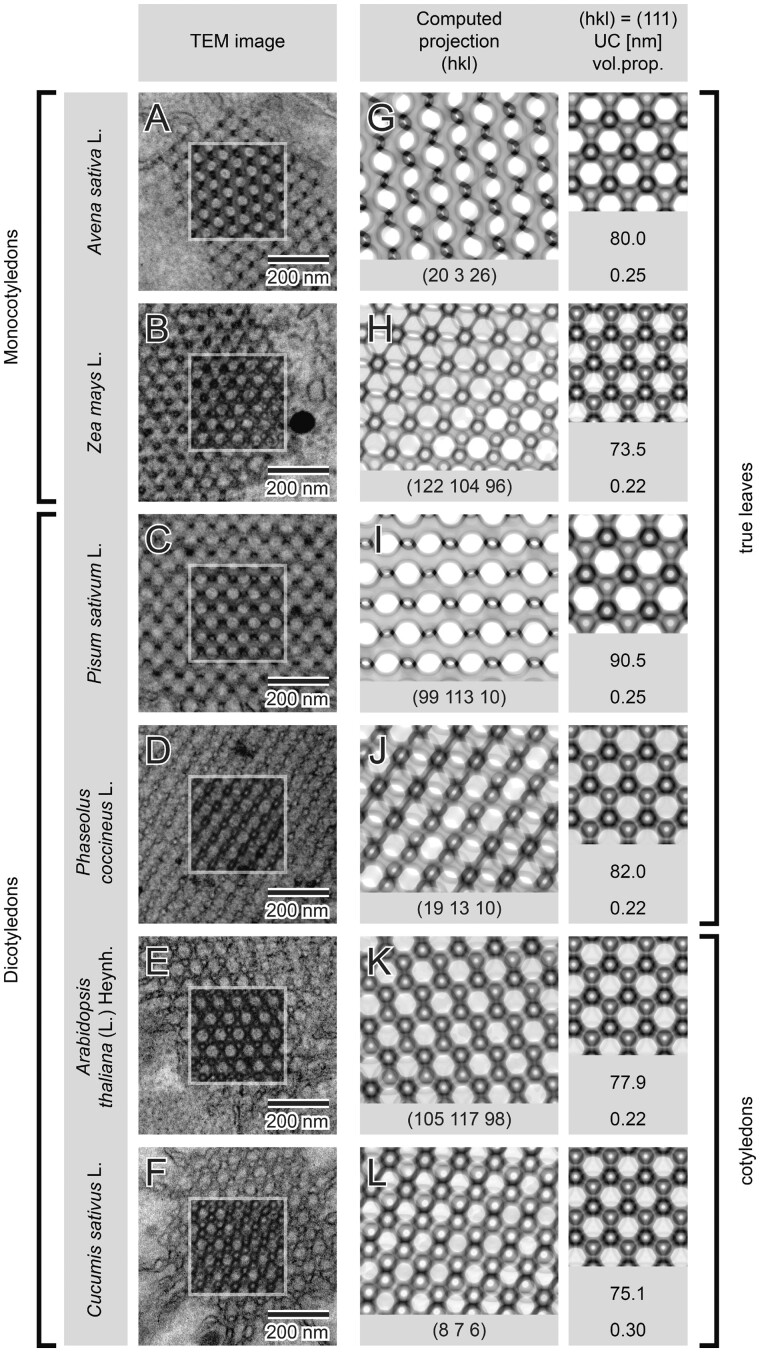
Diamond surface type—a dominating form of the PLB structure in etioplasts of angiosperms. Ultrastructure of the PLB in etiolated seedlings of several species from monocots (A and B) and dicots (C–F) exhibiting hypogeal (A–D) and epigeal (E and F) germination show the diamond type of cubic structure. Regions marked with rectangles present superposition of computed projections and TEM images using multiply blend mode (A–F). Matched computed projections are shown together with insets presenting 111 orientations of particular surfaces (G–L); all parameters used to generate projections are listed in Supplemental Table S1.

**Figure 9:**
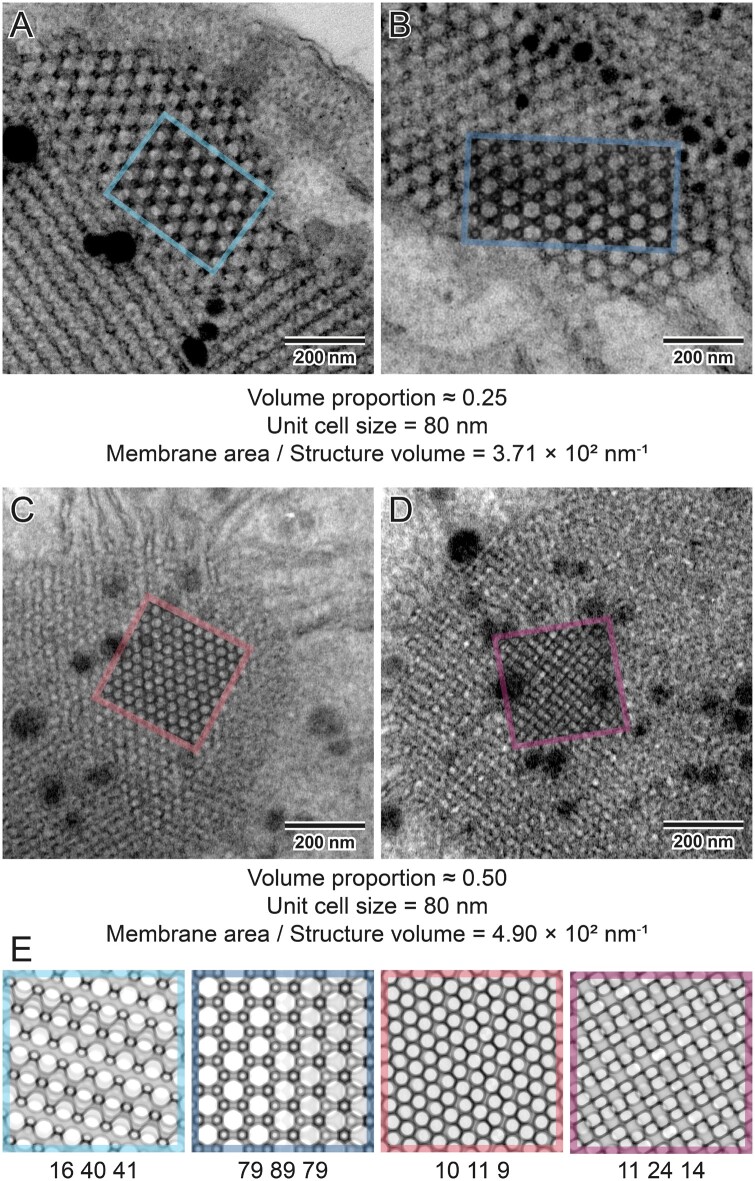
Imbalanced versus balanced nature of PLB cubic membranes. PLBs are cubic membranes of diamond configuration characterized by an imbalanced structure in which two aqueous channels are geometrically different (A and B); regions marked with colored rectangles show a superposition of computed projections and TEM images using multiply blend mode. Changes in the volume proportion resulting in geometrically balanced PLB structure without disturbances in the UC size are observed in plants over accumulating protochlorophyllide (*pif1* mutants of Arabidopsis)—a primary precursor pigment of etioplasts (C and D). Computed projections show differences in the observed patterns of networks that structurally differ only in the volume proportion ratio (E). This variability also influences the structure’s membrane packaging potential; geometrically balanced PLBs can accumulate more membrane components in the given volume; all parameters used to generate projections are listed in Supplemental Table S1.

**Figure 10:**
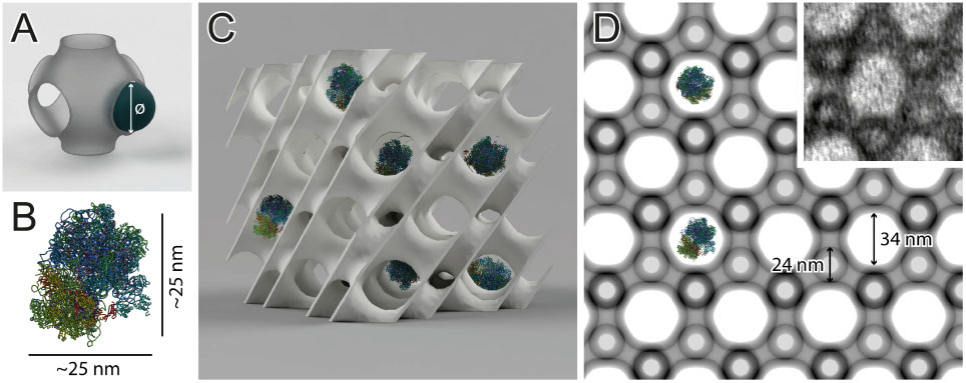
Penetrability of the 3D prolamellar network—combining ultrastructural and molecular data. To estimate the maximum diameter of a sphere that can freely penetrate through a channel of the entire structure, the “percolation threshold” implemented in SPIRE can be computed (A). Here we used an example of plastid ribosome (B, PDB 5MMM, spinach (*Spinacia oleracea*) 70S chloroplast ribosome modeled using Chimera software) whose size is comparable with diameters of the oat PLB channels. Using the percolation threshold function, it is possible to estimate whether a molecule whose spatial structure is already revealed could move freely through the channels of a particular cubic surface (C). The percolation threshold (31.57 nm) of a given network (UC size 80 nm, volume proportion 0.25), identified by a structure’s projection (D), can be calculated in SPIRE. The diamond network of oat PLB in $\left(111\right)$ direction is presented with ribosomes in the same scale, the region marked with a white square presents superposition of computed projection and TEM image using multiply blend mode. Note that proper calculations are also provided for the surfaces recognized by projections in which the channel’s maximal diameter (40.11 nm) is not visible. In this example, ribosome size is smaller than the oat PLB’s percolation limit, which indicates that this molecule can move freely through the larger aqueous channel of the PLB network (stroma) of oat, hypothetically fulfilling its biological function directly inside the cubic structure; all parameters used to generate projection are listed in Supplemental Table S1.

Using SPIRE and the described matching process, we identified the diamond surface type to be a dominating form of PLB cubic structures in the plethora of angiosperm species representing hypo- and epigeal germination as well as mono and dicotyledonous plants (oat (*Avena sativa*): Figures  6–8; pea (*Pisum sativum*), runner bean, cucumber (*Cucumis sativus*), *Arabidopsis thaliana*, and maize (*Zea mays*): Figure  8). Although in all analyzed examples the PLBs matched the diamond surface, the UC size and volume proportion of both aqueous channels varied between 73.5–90.5 nm and 0.2–0.3, respectively (see Supplemental Table S1). It was reported before that, in particular species or growing conditions, PLBs can also adopt an unusual structural type described as an “open PLB” (Gunning, 2001; Rudowska et al., 2012; Skupien et al., 2017). Among all registered PLB micrographs of analyzed plants (100–300/species), we observed such geometry only in ˂2% of visualized oat PLBs. We also showed that other network structural parameters were very similar both within one seedling (Supplemental Figure S6) and in different plants of the same species identically grown (see network parameters for oat in Figures  6–10). Note, however, that the etiolation period can influence PLB structural parameters. We detected that extended time of etiolation resulted in the increase of PLB UC size in oat etioplasts, from 80 nm registered in 1-week etiolated plants up to ∼87.3 after 2 weeks of skotomorphogenesis (Figures  6–10; Supplemental Figure S6).

In specific cases, the PLB can adopt a—within matching accuracy—geometrically balanced diamond structure with a volume proportion reaching 0.5. Such a configuration has been so far identified in mutant plants with a disturbed composition of the PLB membranes only; for example, PHYTOCHROME-INTERACTING FACTOR 1 mutant plants (*pif1*) over-accumulating chlorophyll precursor (protochlorophyllide) (Bykowski et al., 2020; Figure  9). Note that a substantial increase in volume proportion alone, without changes in UC size, causes a rise in the membrane area packed in a given volume (Figure  9). Therefore, when the PLB size is maintained between different genotypes, such a geometrically balanced PLB network might store significantly larger amounts of membrane components, including enzymatic proteins and galactolipids crucial for efficient etioplast–chloroplast transition.

In Figure  10, we present the exemplary utilization of the percolation threshold function. The obtained values indicate that the oat PLB structure enables a free flow of chloroplast ribosome particles through a larger aqueous network channel. Our percolation limit calculation stays in line with recent experimental electron cryo-tomography data, which confirmed the presence of fully assembled ribosomes at a stromal side of a ruptured pea etioplast PLB (Floris and Kuhlbrandt, 2021). However, it should be stressed that in both cases the size of the ribosome and the channel diameter are similar. Therefore, in PLBs of smaller UC size or varying volume proportion registered in different species, such mobility will be blocked. This suggests that ribosome presence in the PLB network may not be crucial for its proper functioning.

## Discussion

In this work, we introduced a software tool (SPIRE), which, based on “nodal surface” models, generates synthetic microscopy images of cubic membranes, bicontinuous phases and other structures. This tool enables the stereological identification of 3D structures based on their 2D projections, a key element in understanding structure–function relationships. We have demonstrated the basic concepts and workflow of SPIRE with a novel application to one of the common examples of cubic membranes occurring in nature—etioplast prolamellar bodies. We revealed that PLB configurations resemble a diamond surface type and, despite earlier assumptions, is not based on a lonsdaleite (wurtzite) structure, at least for the plants analyzed here. Moreover, this work serves as a reference paper for the open-source SPIRE tool.

The development of the interdisciplinary field of naturally occurring cubic structures relies on the availability of tools to analyze observed structures; tools which can be robustly used without a need for an in-depth understanding of the mathematical background.

At this stage, much of the experimental work of biologists does not contribute to the field and might even pass unnoticed due to the lack of a common recognition of cubic arrangements. This is particularly regrettable given that biological bicontinuous structures have achieved a property that remains largely elusive in synthetic cubic phases: structure sizes (lattice parameters) >50 nm.

By expanding the pioneering work by Deng and Mieczkowski (1998), SPIRE fills a gap in the field of surface type identification of cubic membranes, which has only been partially covered by previous methods.

In future work, we aim to improve and extend SPIRE capabilities. A prime target is to automate the matching process of the simulated projection with the actual TEM image. Here the toolbox of image analysis and classification can be employed. A very promising approach here is deep neural networks, specifically convolutional neural networks, which have been proven to efficiently classify images (Krizhevsky et al., 2012; Rawat and Wang, 2017). Having several projections of a structure at different orientations available (as is the case for the samples presented here) could significantly increase the matching accuracy. SPIRE is ideally suited to generate training data sets of nearly arbitrary size for these purposes.

An anticipated broad interest in periodic structure recognition in biological samples enabled by SPIRE will lead to the proper identification of many naturally occurring bicontinuous structures, including geometric measurements computed by the tool. Starting with and extending the tables and data of cubic membrane occurrence in biological systems provided by Almsherqi et al. (2009), Landh (1996) and further literature, this could be the start for a new repository, connecting geometric structures with their natural or synthetic occurrence and functions. Such a database would open opportunities for the meta-analyses of geometric arrangements occurrence in the contexts of their, for example, composition, evolutionary background, developmental importance, and biological meaning, and thus pave the way for insights on broader scales.

In most of the studies, the cubic membranes’ appearance is reported without further interpretation of neither the data nor their numerical analyses. SPIRE could help to re-evaluate and properly annotate numerous already published structures. SPIRE enables acquiring several spatial parameters of the network, which might also be interpreted in the context of other experimental data, for example, percolation limit with the mobility of molecules of given sizes whose presence in the aqueous environment of the network has been confirmed in biochemical studies.

SPIRE is a key tool to accelerate the dynamic field combining actual biological data, computer modeling, and finally, obtaining synthetic periodic structures based on natural ones. Therefore, SPIRE has the potential to broaden our understanding of cellular cubic membranes, their biological role, and their relevance in designing nature-inspired artificial bicontinuous phases of comprehensive utilization.

## Materials and methods

### Implementation details

The tool (https://sourceforge.net/projects/spire-tool/) as well as the source code (https://github.com/tohain/SPIRE) and all dependencies are open source and thus freely and openly available. The software was entirely written in C++ providing an intuitive GUI, shown in Supplemental Figure S7, implemented using the QT libraries (https://doc.qt.io/). Several libraries are used in this tool: integer math library (https://cs.uwaterloo.ca/~astorjoh/iml.html), openblas (https://www.openblas.net/), gnu multiprecision library (https://gmplib.org/), and gnu multiple precision floating point reliable library (https://www.mpfr.org/) are used to compute minimal UCs, zlib (https://www.zlib.net/), and libpng (http://www.libpng.org/pub/png/libpng.html) are used to output projections to the .png image format, the (CGAL (https://www.cgal.org/) is used to reconstruct surfaces to measure their area. Furthermore the algorithms from (Felzenszwalb and Huttenlocher, 2012) are implemented to compute the EDM and the Hoshen and Kopelman (1976) algorithm is used to compute the percolation threshold.

A separation of the computational core code and the interface code allows the use as a library to incorporate into further projects. A simple command line interface for the creation of large batches of projections as an example is included.

The code is designed to allow an easy implementation of further surface types, given in the form of an implicit level-set equation.

### Plant material and growth conditions

Oat (*A.* *sativa* L.), maize (*Z.* *mays* L.), pea (*P.* *sativum* L.), runner bean (*P.* *coccineus* L.), and cucumber (*C.* *sativus* L.) dark-germinated seedlings were etiolated for 1 week in high closed glass containers on wet paper moistened with nutrient solution containing 3 mM Ca(NO_3_)_2_, 1.5 mM KNO_3_, 1.2 mM MgSO_4_, 1.1 mM KH_2_PO_4_, 0.1 mM C_10_H_12_N_2_O_8_FeNa, 5 μM CuSO_4_, 2 μM MnSO_4_ · 5H_2_O, 2 μM ZnSO_4_ · 7H_2_O, and 15 nM (NH_4_)6Mo_7_O_24_ · 4H_2_O, pH 6.0–6.5, room temperature (RT). An additional week of etiolation was applied for oat seedlings used in particular experiments. Seeds of *A.* *thaliana* Col-0 (N1092) and mutant *pif1* (N66041; Huq et al., 2004) were obtained from The European Arabidopsis Stock Center. Seeds were stratified in 4°C for 24 h, and 4 h illumination (120 μmol photons m^−2^ s^−1^ 23°C) was applied to induce germination. Seedlings were etiolated for 5 d in Petri dishes on Murashige and Skoog Basal Medium supplemented with Gamborg B5 vitamin mixture (M0231; Duchefa Biochemie, Haarlem, Netherlands) and 0.8% (w/v) Phytagel (P8169; Sigma-Aldrich, St Louis, MO, USA) in 23°C. Leaf and cotyledon samples were collected under photomorphogenetically inactive dim green light.

### TEM

Leaf specimens were fixed in 2.5% (v/v) glutaraldehyde in 0.05 M cacodylate buffer, pH 7.4 (prepared using 25% v/v glutaraldehyde solution G5882, Sigma-Aldrich; sodium cacodylate trihydrate C0250, Sigma-Aldrich; pH adjusted with 0.1 M HCl) for 2 h, washed, and postfixed in 2% (w/v) OsO_4_ in 0.05 M cacodylate buffer, pH 7.4 (prepared using 4% w/v OsO_4_ solution R1023; Agar Scientific, Essex, UK) at 4°C over-night. Samples were dehydrated in a graded series of acetone and embedded in epoxy resin (AGR1031 Agar 100 Resin Kit, Agar Scientific). The material was cut on a Leica UCT ultramicrotome into 70 nm sections. Samples were analyzed in a JEM 1400 electron microscope (Jeol) equipped with a Morada G2 CCD camera (EMSIS GmbH, Münster, Germany) in the Laboratory of Electron Microscopy, Nencki Institute of Experimental Biology of Polish Academy of Sciences, Warsaw, Poland. The PLB ultrastructural features were measured with the help of ImageJ software (Abramoff et al., 2004). The periodicity of 2D sections was calculated based on averaged values obtained from Fast Fourier Transform of PLB cross-sections. PLB tubule diameters were measured manually based on each tubule’s outer limits in particular orientations of PLB cross-sections.

### In silico image generation and manipulation

Projections obtained using SPIRE were superimposed on TEM micrographs (where applicable) using multiply blend mode in Adobe Photoshop. In Figure  7C, the image was obtained using Adobe Photoshop by deleting a portion of the TEM micrograph and substituting it with projections generated using SPIRE. Random noise was added using Add Noise filter with Gaussian Distribution and Monochromatic settings on a uniform gray image (RGB 127 127 127), blurred using Gaussian blur filter, and superimposed on the image using multiply blend mode. Meshes of 3D models were generated in Houdini using the level-set representation of the surfaces and rendered using Autodesk Fusion 360 software. The 70S chloroplast ribosome was obtained from RCSB PDB (accession number 5MMM) and rendered using UCSF Chimera software (Pettersen et al., 2004).

## Supplemental data

The following materials are available in the online version of this article.

**Supplemental Figure S1.** Choice of lattice vectors of the fundamental UC of the lonsdaleite surface.

**Supplemental Figure S2.** Multi-layer membrane structures and channel enumeration.

**Supplemental Figure S3.** Renderings of the fundamental UCs of the built-in structures.

**Supplemental Figure S4.** Diamond and gyroid type surfaces and computer simulation of TEM images of respective structures cut into sections of variable thickness (40–250 nm).

**Supplemental Figure S5.** Gallery of selected hkl projections of four different surface types implemented in the software.

**Supplemental Figure S6.** PLB network structural parameters are stable in etioplasts of the same seedling.

**Supplemental Figure S7.** Screenshot of the GUI of SPIRE.

**Supplemental Table S1.** Choice of lattice vectors for fundamental UCs.

**Supplemental Table S2.** Parameters used to generate projections.

## Supplementary Material

kiab476_Supplementary_Data

## Notes

T.H., M.B., G.E.S.T., and Ł.K. wrote the original draft; T.H., M.B., M.S., M.E., G.E.S.T., and Ł.K. edited and review the manuscript; T.H. and M.B. designed the software; T.H. implemented the software; T.H. and M.S. designed computational solutions; Ł.K. provided resources (TEM images); M.E., G.E.S.T., and Ł.K. supervised the project, M.B. and Ł.K. performed experiments; M.B. performed image analysis; T.H., M.B., and Ł.K. prepared figures; G.E.S.T. proposed the research; T.H., M.B., G.E.S.T., and Ł.K. provided conception of the project.

The author responsible for distribution of materials integral to the findings presented in this article in accordance with the policy described in the Instructions for Authors (https://academic.oup.com/plphys/pages/general-instructions) is Łucja Kowalewska (lucja.kowalewska@uw.edu.pl).

## References

[kiab476-B1] Abramoff MD , Magalhaes PJ, Ram SJ (2004) Image processing with ImageJ. Biophotonics Int 11**:** 36–42

[kiab476-B2] Adam Z , Charuvi D, Tsabari O, Knopf RR, Reich Z (2011) Biogenesis of thylakoid networks in angiosperms: knowns and unknowns. Plant Mol Biol 76: 221–234 20859754 10.1007/s11103-010-9693-5

[kiab476-B3] Almsherqi ZA , Kohlwein SD, Deng Y (2006) Cubic membranes: a legend beyond the Flatland of cell membrane organization. J Cell Biol 173**:** 839–844 16785319 10.1083/jcb.200603055 PMC2063909

[kiab476-B4] Almsherqi ZA , Landh T, Kohlwein SD, Deng Y (2009) Chapter 6- Cubic membranes: the missing dimension of cell membrane organization. International Review of Cell and Molecular Biology, volume of International Review of Cell and Molecular Biology, Academic Press, Cambridge, MA, pp 275–342 10.1016/S1937-6448(08)02006-6 PMC7105030 19349040

[kiab476-B5] Armarego-Marriott T , Kowalewska Ł, Burgos A, Fischer A, Thiele W, Erban A, Strand D, Kahlau S, Hertle A, Kopka J, et al (2019) Highly resolved systems biology to dissect the etioplast-to-chloroplast transition in tobacco leaves. Plant Physiol 180: 654–681 30862726 10.1104/pp.18.01432 PMC6501100

[kiab476-B6] Bates FS **(** 2005 **)** Network phases in block copolymer melts. MRS Bull 30**:** 525–532

[kiab476-B7] Brakke KA **(** 1992 **)** The surface evolver. Exp Math 1**:** 141–165

[kiab476-B8] Bykowski M , Mazur R, Buszewicz D, Szach J, Mostowska A, Kowalewska L (2020) Spatial nano-morphology of the prolamellar body in etiolated *Arabidopsis thaliana* plants with disturbed pigment and polyprenol composition. Front Cell Dev Biol 8: 586628 33117813 10.3389/fcell.2020.586628 PMC7578251

[kiab476-B9] Callens SJP , Uyttendaele RJC, Fratila-Apachitei LE, Zadpoor AA (2020) Substrate curvature as a cue to guide spatiotemporal cell and tissue organization. Biomaterials 232: 119739 31911284 10.1016/j.biomaterials.2019.119739

[kiab476-B10] Cazzonelli CI , Hou X, Alagoz Y, Rivers J, Dhami N, Lee J, Marri S, Pogson BJ (2020) A cis-carotene derived apocarotenoid regulates etioplast and chloroplast development. eLife 9: e45310 32003746 10.7554/eLife.45310 PMC6994220

[kiab476-B11] Chong K , Deng Y (2012) Chapter 15 - The three dimensionality of cell membranes: lamellar to cubic membrane transition as investigated by electron microscopy. *In* Di Paolo G, Wenk MR, eds, Methods in Cell Biology, Vol. 108 of Lipids, Academic Press, Cambridge, MA, pp 317–343. 10.1016/B978-0-12-386487-1.00015-8 22325609

[kiab476-B12] Cui C , Deng Y, Han L (2020) Bicontinuous cubic phases in biological and artificial self-assembled systems. Sci China Mater 63: 686–702 10.1007/s40843-019-1261-1 PMC7094945 32219007

[kiab476-B13] Da TKF , Cohen-Steiner D (2020) Advancing Front Surface Reconstruction. CGAL Editorial Board, Ed 5.2

[kiab476-B14] Demurtas D , Guichard P, Martiel I, Mezzenga R, H’ebert C, Sagalowicz L (2015) Direct visualization of dispersed lipid bicontinuous cubic phases by cryo-electron tomography. Nat Commun 6**:** 8915 26573367 10.1038/ncomms9915 PMC4660369

[kiab476-B15] Deng Y , Mieczkowski M (1998) Three-dimensional periodic cubic membrane structure in the mitochondria of amoebae *Chaos carolinensis*. Protoplasma 203**:** 16–25

[kiab476-B16] Deng Y , Marko M, Buttle KF, Leith A, Mieczkowski M, Mannella CA (1999) Cubic membrane structure in Amoeba (*Chaos carolinensis*) mitochondria determined by electron microscopic tomography. J Struct Biol 127**:** 231–239 10544048 10.1006/jsbi.1999.4147

[kiab476-B17] Evans ME , Hyde ST (2011) From three-dimensional weavings to swollen corneocytes. J Royal Soc Interface 8: 1274–1280 10.1098/rsif.2010.0722 PMC3140722 21398404

[kiab476-B18] Evans ME , Roth R (2014) Shaping the skin: the interplay of mesoscale geometry and corneocyte swelling. Phys Rev Lett 112: 038102 24484167 10.1103/PhysRevLett.112.038102

[kiab476-B19] Felzenszwalb PF , Huttenlocher DP (2012) Distance transforms of sampled functions. Theory Comput 8: 415–428

[kiab476-B20] Floris D , Kuhlbrandt W (2021) Molecular landscape of etioplast inner membranes in higher plants. Nat Plants 7**:** 514–523 33875833 10.1038/s41477-021-00896-z PMC8055535

[kiab476-B21] Fujii S , Nagata N, Masuda T, Wada H, Kobayashi K (2019) Galactolipids are essential for internal 617 membrane transformation during etioplast-to-chloroplast differentiation. Plant Cell Physiol 60**:** 1224–1238 30892620 10.1093/pcp/pcz041 PMC6553665

[kiab476-B22] Gunning BE , Steer MW (1975) Ultrastructure and the Biology of Plant Cells, Arnold, London

[kiab476-B23] Gunning BES (2001) Membrane geometry of” open” prolamellar bodies. Protoplasma 215: 4–15 11732064 10.1007/BF01280299

[kiab476-B24] Han L , Fujita N, Chen H, Jin C, Terasaki O, Che S (2020) Crystal twinning of bicontinuous cubic structures. IUCrJ 7: 228–237 10.1107/S2052252519017287 PMC7055389 32148851

[kiab476-B25] Hoshen J , Kopelman R (1976) Percolation and cluster distribution. I. Cluster multiple labeling technique and critical concentration algorithm. Phys Rev B 14: 3438–3445

[kiab476-B26] Huq E , Al-Sady B, Hudson M, Kim C, Apel K, Quail PH (2004) PHYTOCHROME-INTERACTING FACTOR 1 is a critical bHLH regulator of chlorophyll biosynthesis. Science 305: 1937–1941 15448264 10.1126/science.1099728

[kiab476-B27] Hyde S , Blum Z, Landh T, Lidin S, Ninham BW, Andersson S, Larsson K (1996) The Language of Shape: The Role of Curvature in Condensed Matter: Physics, Chemistry and Biology, Ed 1, Elsevier Science, Amsterdam, Netherlands; New York, NY

[kiab476-B28] Ikeda T (1968) Analytical studies on structure of prolamellar body. Bot Mag.-TOKYO 81: 517

[kiab476-B29] Jarsch IK , Daste F, Gallop JL (2016) Membrane curvature in cell biology: an integration of molecular mechanisms. J Cell Biol 214**:** 375–387 27528656 10.1083/jcb.201604003 PMC4987295

[kiab476-B30] Kirkensgaard JJK , Fragouli P, Hadjichristidis N, Mortensen K (2011) Perforated lamellae morphology in novel P2VP(PDMS-b-PI-b-PS)2 3-miktoarm star qarterpolymer. Macromolecules 44**:** 575–582

[kiab476-B31] Kittel C (2004) Introduction to Solid State Physics, Ed 8, Wiley, Hoboken, NJ

[kiab476-B32] Klinowski J , Mackay AL, Terrones H, Klinowski J, Mackay AL (1996) Curved surfaces in chemical structure. Philos Trans Royal Soc Lond A Mathe Phys Eng Sci 354**:** 1975–1987

[kiab476-B33] Kowalewska Ł , Mazur R, Suski S, Garstka M, Mostowska A (2016) Three-dimensional visualization of the tubular-lamellar transformation of the internal plastid membrane network during runner bean chloroplast biogenesis. Plant Cell 28**:** 875–891 27002023 10.1105/tpc.15.01053 PMC4863387

[kiab476-B34] Kowalewska Ł , Bykowski M, Mostowska A (2019) Spatial organization of thylakoid network in higher plants. Bot Lett 166: 326–343

[kiab476-B35] Kozlov MM , Campelo F, Liska N, Chernomordik LV, Marrink SJ, McMahon HT (2014) Mechanisms shaping cell membranes. Curr Opin Cell Biol 29**:** 53–60 24747171 10.1016/j.ceb.2014.03.006 PMC4180517

[kiab476-B36] Krizhevsky A , Sutskever I, Hinton GE (2012) ImageNet classification with deep convolutional neural networks. Proceedings of the 25th International Conference on Neural Information Processing Systems, Vol. 1, NIPS’12, Curran Associates Inc., Red Hook, NY, pp 1097–1105

[kiab476-B37] Landh T (1996) Cubic cell membrane architectures. Taking another look at membrane bound cell spaces. PhD thesis. Department of Food Technology, Lund University, Lund, Sweden

[kiab476-B38] Lou HY , Zhao W, Zeng Y, Cui B **(** 2018 **)** The role of membrane curvature in nanoscale topography- induced intracellular signaling. Account Chem Res 51**:** 1046–1053 10.1021/acs.accounts.7b00594 PMC7384307 29648779

[kiab476-B39] Luzzati V (1997) Biological significance of lipid polymorphism: the cubic phases. Curr Opin Struct Biol 7**:** 661–668 9345624 10.1016/s0959-440x(97)80075-9

[kiab476-B40] Menke W (1963) Zur Stereometrie der Heitz-Leyonschen Kristalle von Chlorophytum comosum. Z Für Naturforsch B 18**:** 821–826

[kiab476-B41] Mezzenga R , Seddon JM, Drummond CJ, Boyd BJ, Schr¨oder-Turk GE, Sagalowicz L (2019) Nature-inspired design and application of lipidic lyotropic liquid crystals. Adv Mater 31**:** 1900818 10.1002/adma.201900818 31222858

[kiab476-B42] Mickel W , Munster S, Jawerth LM, Vader DA, Weitz DA, Sheppard AP, Mecke K, Fabry B, Schroder-Turk GE (2008) Robust pore size analysis of filamentous networks from three-dimensional confocal microscopy. Biophys J 95**:** 6072–6080 18835899 10.1529/biophysj.108.135939 PMC2599830

[kiab476-B43] Nguyen HC , Melo AA, Kruk J, Frost A, Gabruk M (2021) Photocatalytic LPOR forms helical lattices that shape membranes for chlorophyll synthesis. Nat Plants 7**:** 437–444 33875834 10.1038/s41477-021-00885-2

[kiab476-B44] Norlen L , Al-Amoudi A (2004) Stratum corneum keratin structure, function, and formation: the cubic rod-packing and membrane templating model. J Invest Dermatol 123: 715–732 15373777 10.1111/j.0022-202X.2004.23213.x

[kiab476-B45] O’Keeffe M , Plevert J, Teshima Y, Watanabe Y, Ogama T (2001) The invariant cubic rod (cylinder) packings: symmetries and coordinates. Acta Cryst A 57**:** 110–111 11124509 10.1107/s010876730001151x

[kiab476-B46] Pettersen EF , Goddard TD, Huang CC, Couch GS, Greenblatt DM, Meng EC, Ferrin TE (2004) UCSF Chimera—A visualization system for exploratory research and analysis. J Comput Chem 25**:** 1605–1612 15264254 10.1002/jcc.20084

[kiab476-B47] Pipitone R , Eicke S, Pfister B, Glauser G, Falconet D, Uwizeye C, Pralon T, Zeeman SC, Kessler F, Demarsy E (2021) A multifaceted analysis reveals two distinct phases of chloroplast biogenesis during de-etiolation in Arabidopsis. eLife 10: e62709 33629953 10.7554/eLife.62709 PMC7906606

[kiab476-B48] Pribil M , Labs M, Leister D **(** 2014 **)** Structure and dynamics of thylakoids in land plants. J Exp Bot 65**:** 1955–1972 24622954 10.1093/jxb/eru090

[kiab476-B49] Rawat W , Wang Z (2017) Deep convolutional neural networks for image classification: a comprehensive review. Neural Comput 29**:** 2352–2449 28599112 10.1162/NECO_a_00990

[kiab476-B50] Rudowska Ł , Gieczewska K, Mazur R, Garstka M, Mostowska A (2012) Chloroplast biogenesis correlation between structure and function. Biochim Biophys Acta 1817: 1380–1387 22465024 10.1016/j.bbabio.2012.03.013

[kiab476-B51] Sandor A , Fricker MD, Kriechbaumer V, Sweetlove LJ (2021) IntEResting structures: formation and applications of organised smooth endoplasmic reticulum in plant cells. Plant Physiol 185: 550–561 33822222 10.1104/pp.20.00719 PMC8892044

[kiab476-B52] Schoen AH (1970) Infinite periodic minimal surfaces without self-intersections. NASA Technical Note, NASA TN D-5541. National Aeronautics and Space Administration, Springfield, VA

[kiab476-B53] Schroder-Turk GE , Wickham S, Averdunk H, Brink F, Fitz Gerald JD, Poladian L, Large MCJ, Hyde ST (2011) The chiral structure of porous chitin within the wing-scales of Callophrys rubi. J Struct Biol 174**:** 290–295 21272646 10.1016/j.jsb.2011.01.004

[kiab476-B54] Simunovic M , Evergren E, Callan-Jones A, Bassereau P (2019) Curving cells inside and out: roles of BAR domain proteins in membrane shaping and its cellular implications. Annu Rev Cell Dev Biol 35**:** 111–129 31340125 10.1146/annurev-cellbio-100617-060558

[kiab476-B55] Skupień J , Wójtowicz J, Kowalewska Ł, Mazur R, Garstka M, Gieczewska K, Mostowska A (2017) 714 Dark-chilling induces substantial structural changes and modifies galactolipid and carotenoid composition during chloroplast biogenesis in cucumber (*Cucumis sativus* l.) cotyledons. Plant Physiol Biochem 111**:** 107–118 27915172 10.1016/j.plaphy.2016.11.022

[kiab476-B56] Sperling U , Franck F, van Cleve B, Frick G, Apel K, Armstrong GA (1998) Etioplast differentiation in Arabidopsis: both PORA and PORB restore the prolamellar body and photoactive protochlorophyllide–F655 to the cop1 photomorphogenic mutant. Plant Cell 10**:** 283–296 9490750 10.1105/tpc.10.2.283 PMC143981

[kiab476-B57] Stauffer D , Aharony A (1992) Introduction To Percolation Theory, Ed 2, Taylor & Francis, London

[kiab476-B58] **The CGAL Project** **(**2020**)** CGAL User and Reference Manual. CGAL Editorial Board, Ed 5.2

[kiab476-B59] von Schnering HG , Nesper R (1991) Nodal surfaces of Fourier series: fundamental invariants of structured matter. Z Physik B – Condensed Matter 83: 407–412 doi: 10.1007/BF01313411

[kiab476-B60] Wietrzynski W , Engel BD (2021) Chlorophyll biogenesis sees the light. Nat Plants 7**:** 380–381 33875836 10.1038/s41477-021-00900-6

[kiab476-B61] Winter B , Butz B, Dieker C, Schroder-Turk GE, Mecke K, Spiecker E (2015) Coexistence of both gyroid chiralities in individual butterfly wing scales of Callophrys rubi. Proc Natl Acad Sci USA 112: 12911–12916 26438839 10.1073/pnas.1511354112 PMC4620911

[kiab476-B62] Wohlgemuth M , Yufa N, Hoffman J, Thomas EL (2001) Triply periodic bicontinuous cubic microdomain morphologies by symmetries. Macromolecules 34**:** 6083–6089

[kiab476-B63] Yoshioka S , Fujita H, Kinoshita S, Matsuhana B (2014) Alignment of crystal orientations of the multi-domain photonic crystals in Parides sesostris wing scales. J Royal Soc Interface 11**:** 20131029 10.1098/rsif.2013.1029 PMC3899871 24352678

[kiab476-B64] Zhan T , Lv W, Deng Y (2017) Multilayer gyroid cubic membrane organization in green alga Zygnema. Protoplasma 254**:** 1923–1930 28176001 10.1007/s00709-017-1083-2

