# Supplementary material for: SPIRE—a software tool for bicontinuous phase recognition: application for plastid cubic membranes
Source: Plant Physiol. 2021 Oct 18;188(1):81–96. doi: 10.1093/plphys/kiab476 (PMC8774748; doi:10.1093/plphys/kiab476)
Supplement: kiab476_Supplementary_Data [file kiab476_supplementary_data.pdf]

Supplementary Material for SPIRE, a software tool for bicontinuous phase recognition: application for plastid cubic membranes

Tobias M. Hain[1,2,3], Michał Bykowski[4], Matthias Saba[5], Myfanwy E. Evans[1], Gerd E. Schröder-Turk[2,6], Łucja Kowalewska[4]

[1]Institute of Mathematics, University of Potsdam, Karl-Liebknecht-Str. 24-25, D-14476 Potsdam OT Golm, Germany

[2]Murdoch University, College of Science, Health, Engineering and Education, Mathematics and Statistics, 90 South St., Murdoch, WA 6150, Australia

[3]Physical Chemistry, Center for Chemistry and Chemical Engineering, Lund University, Lund 22100, Sweden

[4]Department of Plant Anatomy and Cytology, Institute of Experimental Plant Biology and Biotechnology, Faculty of Biology, University of Warsaw, Warsaw, Poland

[5]Adolphe Merkle Institute, University of Fribourg, Chemin des Verdiers 4, CH-1700 Fribourg, Switzerland

[6]The Australian National University, Research School of Physics, Department of Applied Mathematics, Canberra Acton ACT 2601, Australia

.1 Supplementary figures

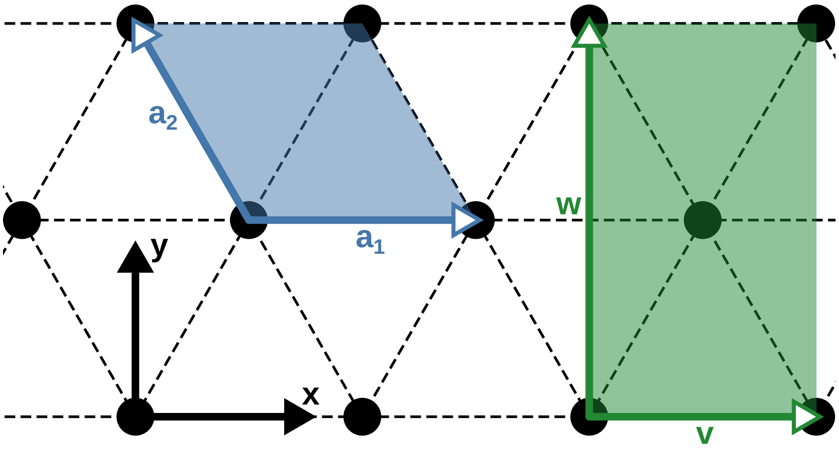

Supplemental Figure S1: **Choice of lattice vectors of the fundamental unit cell of the lonsdaleite surface.** Shown is a top-down view of a hexagonal structure with the canonical choice of the unit cell (lattice vectors a_1 and a_2) and a rectangular unit cell (lattice vectors v and w). For convenience, we chose the rectangular unit cell over the canonical choice. The exact dimensions of the fundamental unit cell are provided in Table S1

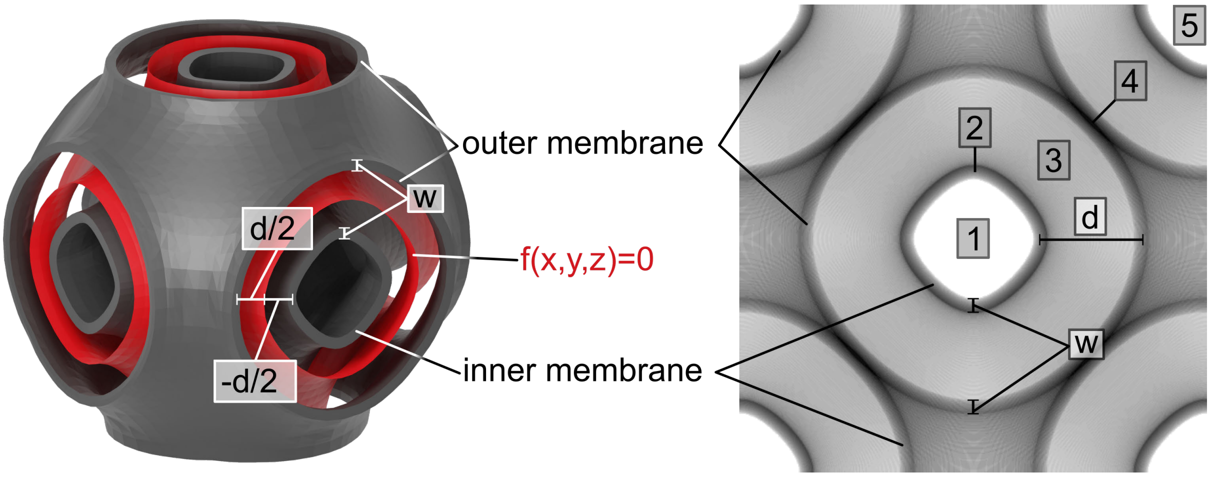

Supplemental Figure S2: **Multi-layer membrane structures and channel enumeration** A primitive surface multi-layer membrane system and its 2D projection in (100) orientation with two membranes of width w at a distance of d computed as parallel surfaces from the level-set membrane, the minimal surface at $f(x, y, z) = 0$, shown in red. The latter is only computed internally and does not show in the projection. The inner membrane is inside of the level-set membrane, therefore has a negative distance. The numbers denote the channel numbers of a total of 5 channels, of which 3 are “true” channels and 2 are membranes, also considered as channels internally.

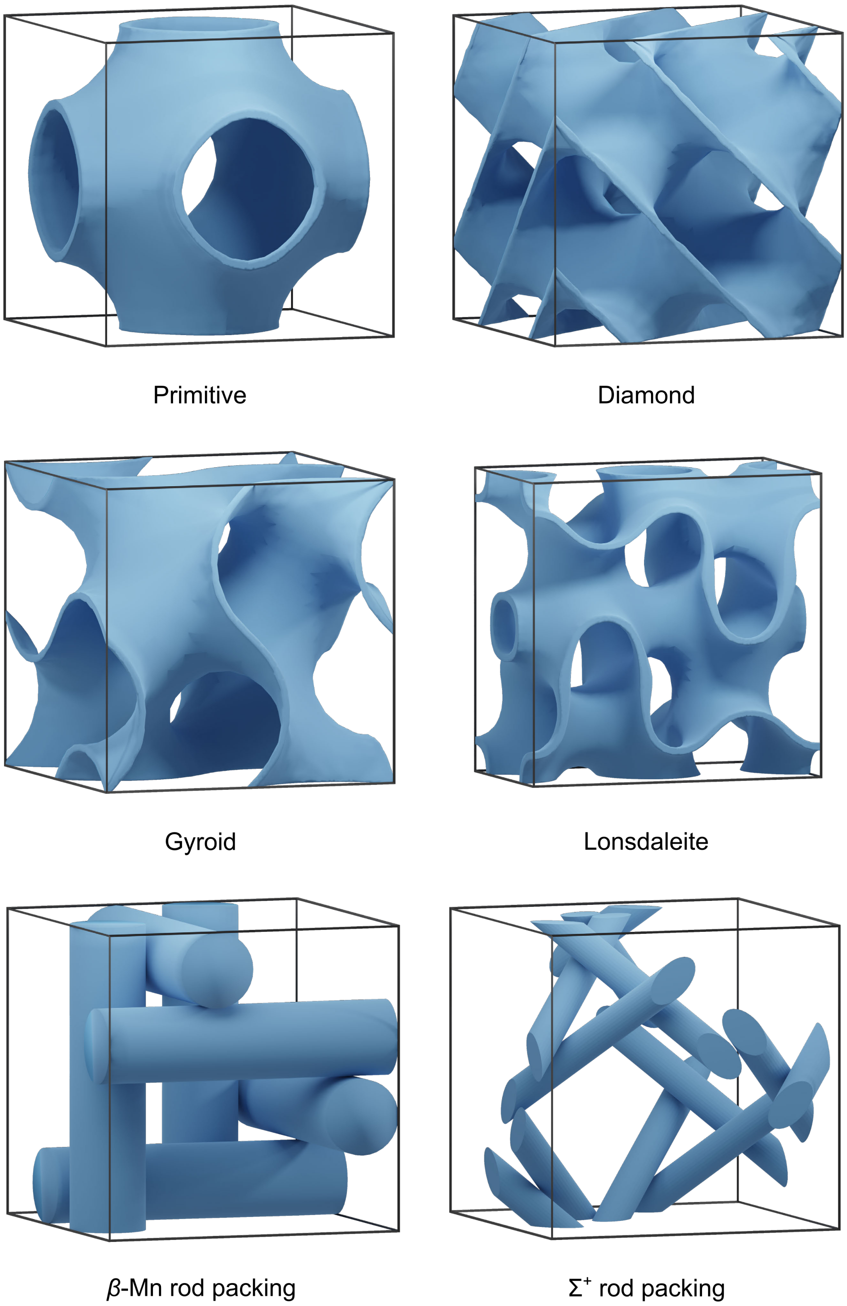

Supplemental Figure S3: **Renderings of the fundamental unit cells of the built-in structures**

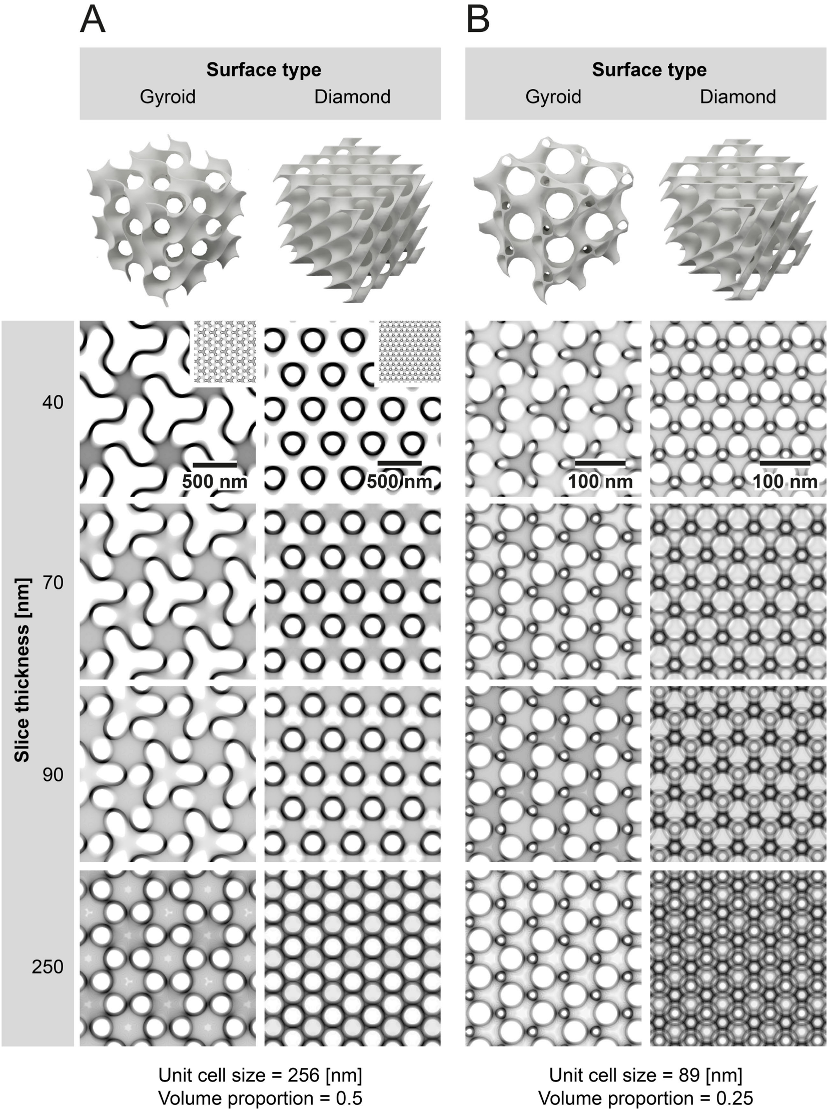

Supplemental Figure S4: **Diamond and gyroid type surfaces and computer simulation of Transmission Electron Microscopy (TEM) images of respective structures cut into sections of variable thickness (40–250** nm**)** The first row presents three-dimensional (3D) models of eight unit cells (UCs) of gyroid and diamond surfaces with balanced (**A**) and imbalanced (**B**) channel proportion. Computer simulations of TEM images are based on structural parameters (UC size and volume proportion – see bottom of the image) of cubic membranes recognized in (**A**) – gyroid of *Zygnema* sp. chloroplasts (Zhan et al., 2017) and (**B**) – diamond of bean *P. coccineus* etioplasts (Kowalewska et al., 2016). Note that for a better comparison, both surface types are simulated using the same structural parameters and are presented in (111) direction only. The figure shows how the slice thickness (subsequent rows) and structure scale (large (**A**) vs. small (**B**)) influence the pattern observed in computer simulations and, therefore, actual TEM images of such structures. Insets visible in the upper right corners of the second row of panel (**A**) present projections from panel (**B**) scaled equally; all parameters used to generate projections are listed in Table S2.

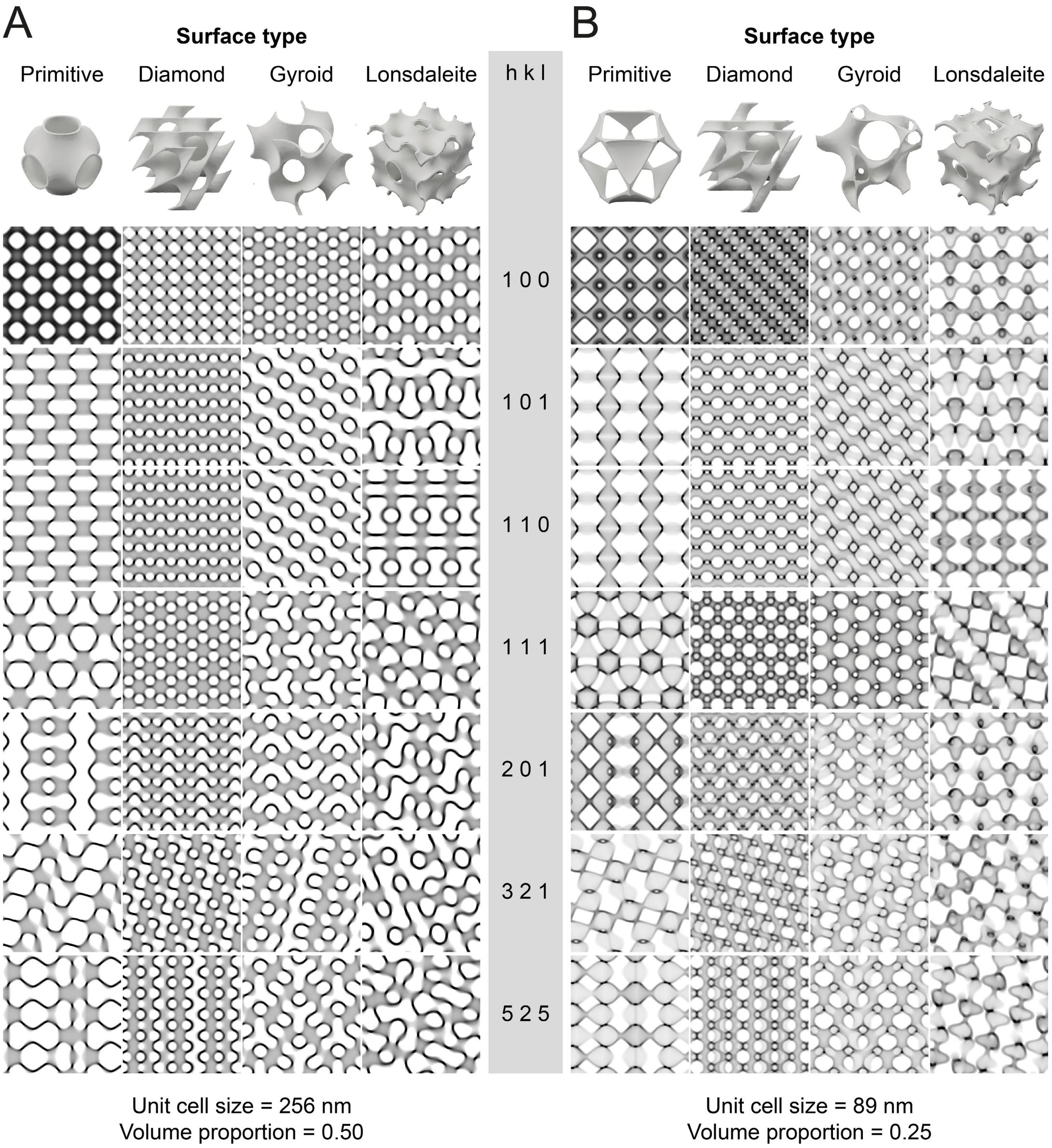

Supplemental Figure S5: **Gallery of selected (*hkl*) projections of four different surface types implemented in the software.** The efficient process of surface matching is preceded by obtaining structural parameters such as unit cell (UC) size and volume proportion estimated directly from Transmission Electron Microscopy (TEM) images. The second step is completed by selecting a proper surface type and recognizing the structure's orientation on the particular micrograph. For this purpose, a basic gallery showing variable (*hkl*) projections of different surface types, based on the idea provided by (Deng and Mieczkowski, 1998), computed for balanced/imbalanced and large/small length scaled structures is a good starting point. Custom, more tailored galleries can be created by the user with the bulk creation function of the tool. Note that projections were computed to simulated TEM samples of 70 nm thickness for membrane structural parameters (UC size and volume proportion), same as in Figure S4 on panels (**A**) and (**B**), respectively. three-dimensional (3D) models of periodic surfaces (first row) are presented for a single UC of all surface types; computed projections of TEM images are scaled to show the same number of UC despite the structure's length scale; all parameters used to generate projections are listed in Table S2.

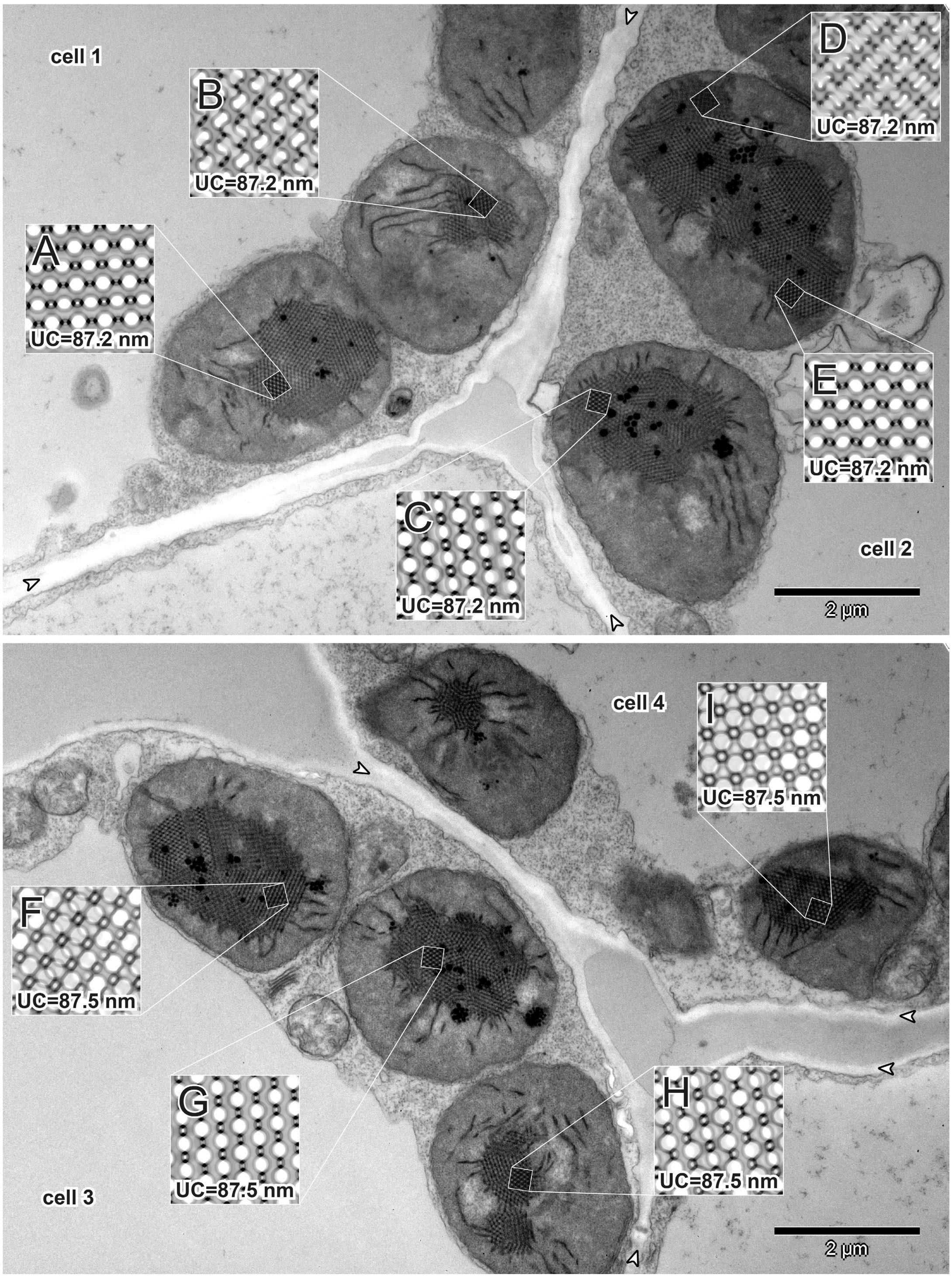

Supplemental Figure S6: **Prolamellar body (PLB) network structural parameters are stable in etioplasts of the same seedling.** Electron micrographs of mesophyll cells in two-week etiolated oat seedlings (same plant) showing etioplasts with diamond-type PLBs (**A**–**H**). PLB surface type was recognized via matching with computed projections of diamond surface type. Regions marked with rectangles present superposition of computed projections and TEM images using multiply blend mode; Regions marked with rectangles present superposition of computed projections and TEM images using multiply blend mode.

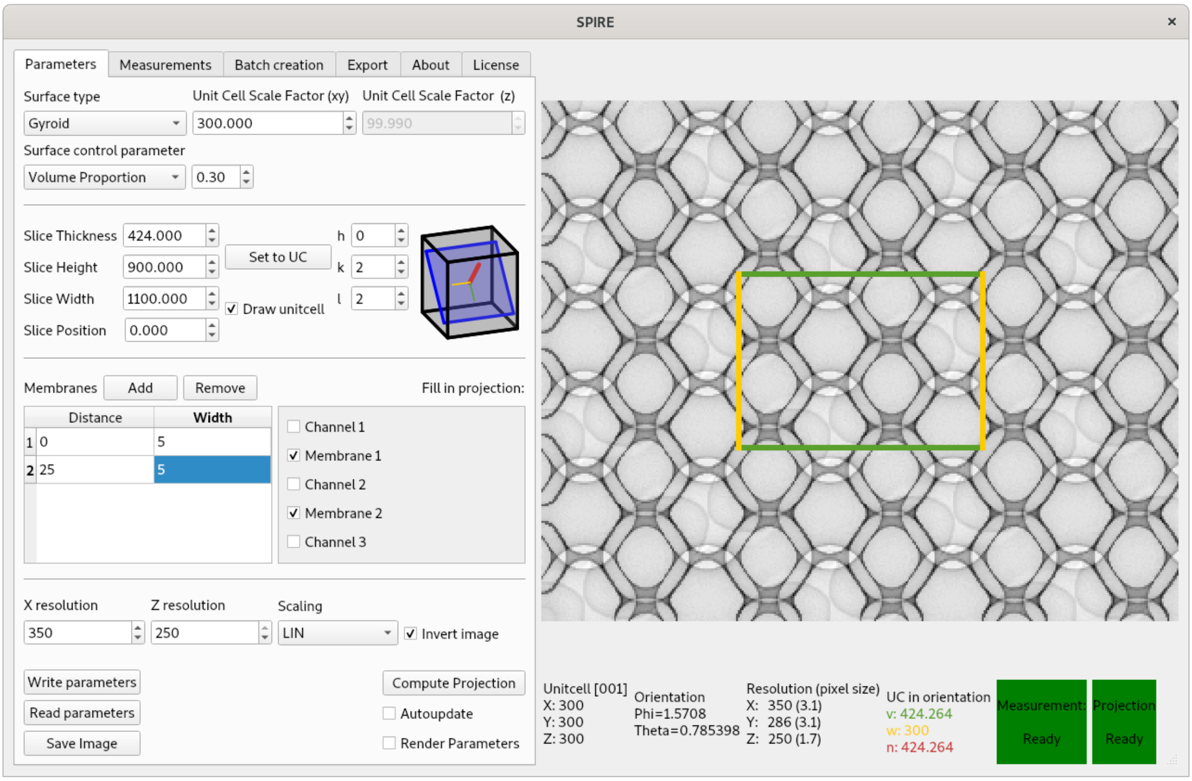

Supplemental Figure S7: **Screenshot of the Graphical User Interface (GUI) of SPIRE**

.2 Supplementary tables

Supplemental Table S1: Choice of lattice vectors for fundamental unit cells

Primitive	$a_1 = (1,0,0)^\intercal$, $a_2 = (0,1,0)^\intercal$, $a_3 = (0,0,1)^\intercal$
Diamond	$a_1 = (1,0,0)^\intercal$, $a_2 = (0,1,0)^\intercal$, $a_3 = (0,0,1)^\intercal$
Gyroid	$a_1 = (1,0,0)^\intercal$, $a_2 = (0,1,0)^\intercal$, $a_3 = (0,0,1)^\intercal$
Lonsdaleite	$a_1 = (1,0,0)^\intercal$, $a_2 = (0,\sqrt{3},0)^\intercal$, $a_3 = (0,0,1.732692)^\intercal$
Beta rod packing	$a_1 = (1,0,0)^\intercal$, $a_2 = (0,1,0)^\intercal$, $a_3 = (0,0,1)^\intercal$
Sigma rod packing	$a_1 = (1,0,0)^\intercal$, $a_2 = (0,1,0)^\intercal$, $a_3 = (0,0,1)^\intercal$

Supplemental Table S2: Parameters used to generate projections shown across the article including those matching actual TEM biological data.

Structure type	UC Scale	Vol. prop.	Slice dimensions [nm]	Slice position [nm]	Orientation	Membrane width [nm]
			Figure 6 F			
Diamond	80	0.25	350 x 350 x 70	200	(5 3 5)	7
			Figure 6 G			
Diamond	80	0.25	350 x 350 x 70	140	(10 13 10)	7
	80	0.25	350 x 350 x 70	140	(5 3 7)	7
			Figure 6 H			
Diamond	80	0.25	350 x 350 x 70	60	(5 3 5)	7
	80	0.25	350 x 350 x 70	60	(2 3 2)	7
			Figure 7 B, C			
Diamond	80	0.25	350 x 350 x 40	190	(18 4 1)	5
	80	0.25	350 x 350 x 40	190	(83 100 107)	5
	80	0.25	350 x 350 x 40	190	(18 4 1)	5
	80	0.25	350 x 350 x 40	190	(79 26 70)	5
	80	0.25	350 x 350 x 40	190	(90 100 11)	5
	80	0.25	350 x 350 x 40	190	(18 4 1)	5
	80	0.25	350 x 350 x 40	190	(45 50 54)	5
	80	0.25	350 x 350 x 40	190	(18 4 1)	5
	80	0.25	350 x 350 x 40	190	(90 100 11)	5
	80	0.25	350 x 350 x 40	190	(79 26 70)	5
	80	0.25	350 x 350 x 40	190	(4 1 1)	5
			Figure 8 A, G			
Diamond	80	0.25	300 x 300 x 80	0	(20 3 26)	7
			Figure 8 B, H			
Diamond	73.5	0.22	300 x 300 x 80	313	(122 104 96)	8
			Figure 8 C, I			
Diamond	90.5	0.25	300 x 300 x 70	275	(99 113 10)	8
			Figure 8 D, J			
Diamond	82	0.22	300 x 300 x 80	55	(19 13 10)	8

			Figure 8 E, K			
Diamond	77.9	0.22	300 x 300 x 80	50	(105 117 98)	8
			Figure 8 F, L			
Diamond	75.1	0.3	300 x 300 x 80	50	(8 7 6)	8
			Figure 9 A			
Diamond	80	0.25	350 x 350 x 70	57	(16 40 41)	5
			Figure 9 B			
Diamond	80	0.22	350 x 350 x 70	58	(79 89 79)	5
			Figure 9 C			
Diamond	80	0.48	350 x 350 x 70	50	(10 11 9)	5
			Figure 9 D			
Diamond	80	0.45	350 x 350 x 70	50	(11 24 14)	5
			Figure 10 D			
Diamond	80	0.25	120 x 240 x 60	195	(1 1 1)	5
			Figure S4 A			
Gyroid	256	0.5	800 x 800 x 40	400	(1 1 1)	15
	256	0.5	800 x 800 x 70	400	(1 1 1)	15
	256	0.5	800 x 800 x 90	400	(1 1 1)	15
	256	0.5	800 x 800 x 250	400	(1 1 1)	15
Diamond	256	0.5	800 x 800 x 40	400	(1 1 1)	15
	256	0.5	800 x 800 x 70	400	(1 1 1)	15
	256	0.5	800 x 800 x 90	400	(1 1 1)	15
	256	0.5	800 x 800 x 250	400	(1 1 1)	15
			Figure S4 B			
Gyroid	89	0.25	350 x 350 x 40	175	(1 1 1)	5
	89	0.25	350 x 350 x 70	175	(1 1 1)	5
	89	0.25	350 x 350 x 90	175	(1 1 1)	5
	89	0.25	350 x 350 x 250	175	(1 1 1)	5
Diamond	89	0.25	350 x 350 x 40	175	(1 1 1)	5
	89	0.25	350 x 350 x 70	175	(1 1 1)	5
	89	0.25	350 x 350 x 90	175	(1 1 1)	5
	89	0.25	350 x 350 x 250	175	(1 1 1)	5
			Figure S5 A			
Primitive	256	0.5	1006 x 1006 x 70	60	(1 0 0)	15
	256	0.5	1006 x 1006 x 70	60	(1 0 1)	15
	256	0.5	1006 x 1006 x 70	60	(1 1 0)	15
	256	0.5	1006 x 1006 x 70	60	(1 1 1)	15
	256	0.5	1006 x 1006 x 70	60	(2 0 1)	15
	256	0.5	1006 x 1006 x 70	60	(3 2 1)	15
	256	0.5	1006 x 1006 x 70	60	(5 2 5)	15
Diamond	256	0.5	1006 x 1006 x 70	60	(1 0 0)	15
	256	0.5	1006 x 1006 x 70	60	(1 0 1)	15
	256	0.5	1006 x 1006 x 70	60	(1 1 0)	15
	256	0.5	1006 x 1006 x 70	60	(1 1 1)	15
	256	0.5	1006 x 1006 x 70	60	(2 0 1)	15

	256	0.5	1006 x 1006 x 70	60	(3 2 1)	15
	256	0.5	1006 x 1006 x 70	60	(5 2 5)	15
Gyroid	256	0.5	1006 x 1006 x 70	60	(1 0 0)	15
	256	0.5	1006 x 1006 x 70	60	(1 0 1)	15
	256	0.5	1006 x 1006 x 70	60	(1 1 0)	15
	256	0.5	1006 x 1006 x 70	60	(1 1 1)	15
	256	0.5	1006 x 1006 x 70	60	(2 0 1)	15
	256	0.5	1006 x 1006 x 70	60	(3 2 1)	15
	256	0.5	1006 x 1006 x 70	60	(5 2 5)	15
Lonsdaleite	256	0.5	1006 x 1006 x 70	60	(1 0 0)	15
	256	0.5	1006 x 1006 x 70	60	(1 0 1)	15
	256	0.5	1006 x 1006 x 70	60	(1 1 0)	15
	256	0.5	1006 x 1006 x 70	60	(1 1 1)	15
	256	0.5	1006 x 1006 x 70	60	(2 0 1)	15
	256	0.5	1006 x 1006 x 70	60	(3 2 1)	15
	256	0.5	1006 x 1006 x 70	60	(5 2 5)	15
Figure S5 B						
Primitive	89	0.25	350 x 350 x 70	60	(1 0 0)	5
	89	0.25	350 x 350 x 70	60	(1 0 1)	5
	89	0.25	350 x 350 x 70	60	(1 1 0)	5
	89	0.25	350 x 350 x 70	60	(1 1 1)	5
	89	0.25	350 x 350 x 70	60	(2 0 1)	5
	89	0.25	350 x 350 x 70	60	(3 2 1)	5
	89	0.25	350 x 350 x 70	60	(5 2 5)	5
Diamond	89	0.25	350 x 350 x 70	60	(1 0 0)	5
	89	0.25	350 x 350 x 70	60	(1 0 1)	5
	89	0.25	350 x 350 x 70	60	(1 1 0)	5
	89	0.25	350 x 350 x 70	60	(1 1 1)	5
	89	0.25	350 x 350 x 70	60	(2 0 1)	5
	89	0.25	350 x 350 x 70	60	(3 2 1)	5
	89	0.25	350 x 350 x 70	60	(5 2 5)	5
Gyroid	89	0.25	350 x 350 x 70	60	(1 0 0)	5
	89	0.25	350 x 350 x 70	60	(1 0 1)	5
	89	0.25	350 x 350 x 70	60	(1 1 0)	5
	89	0.25	350 x 350 x 70	60	(1 1 1)	5
	89	0.25	350 x 350 x 70	60	(2 0 1)	5
	89	0.25	350 x 350 x 70	60	(3 2 1)	5
	89	0.25	350 x 350 x 70	60	(5 2 5)	5
Lonsdaleite	89	0.25	350 x 350 x 70	60	(1 0 0)	5
	89	0.25	350 x 350 x 70	60	(1 0 1)	5
	89	0.25	350 x 350 x 70	60	(1 1 0)	5
	89	0.25	350 x 350 x 70	60	(1 1 1)	5
	89	0.25	350 x 350 x 70	60	(2 0 1)	5
	89	0.25	350 x 350 x 70	60	(3 2 1)	5
	89	0.25	350 x 350 x 70	60	(5 2 5)	5
Figure S6 A						
Diamond	87.2	0.25	300 x 300 x 70	25	(91 13 92)	7
Figure S6 B						
Diamond	87.2	0.25	300 x 300 x 70	25	(18 3 7)	7
Figure S6 C						

Diamond	87.2	0.25	300 x 300 x 70	25	(71 19 72)	7
Figure S6 D						
Diamond	87.2	0.25	300 x 300 x 70	25	(181 25 11)	7
Figure S6 E						
Diamond	87.2	0.25	300 x 300 x 70	25	(90 111 7)	7
Figure S6 F						
Diamond	87.5	0.25	300 x 300 x 70	25	(33 19 22)	7
Figure S6 G						
Diamond	87.5	0.25	300 x 300 x 70	25	(78 9 75)	7
Figure S6 H						
Diamond	87.5	0.25	300 x 300 x 70	25	(81 28 74)	7
Figure S6 I						
Diamond	87.5	0.25	300 x 300 x 70	25	(89 89 76)	7
